# NatD promotes glioblastoma progression through epigenetic activation of FGFR4 signalling

**DOI:** 10.1038/s12276-026-01818-9

**Published:** 2026-09-02

**Authors:** Chan-Woo Kim, Taek-Yeol Jung, Chanil Kim, EungHan Kim, Heui Og Kim, Jiyeon Chae, Hyelee Kim, Ga-Yeon Ko, You-Jung Yeo, Ji-Young Park, Jaehyuk Pyo, Jeong Taeg Seo, Dokyeong Kim, Junseong Park, Seo Jin Kim, Minji An, Seok-Gu Kang, Jong Bae Park, Chul Hoon Kim, Seung-Hyun Jung, Sug Hyung Lee, Ho-Shik Kim, Hyun-Seok Kim

**Affiliations:** 1 Department of Medical Sciences, Graduate School of The Catholic University of Korea, Seoul, Republic of Korea; 2 Department of Biochemistry, College of Medicine, The Catholic University of Korea, Seoul, Republic of Korea; 3 Department of Life Science, College of Natural Science, Ewha Womans University, Seoul, Republic of Korea; 4 OmixAI, Seoul, Republic of Korea; 5 Department of Life Science, College of Natural Science, Sogang University, Seoul, Republic of Korea; 6 Department of Pharmacology, Graduate School of Medical Science, Brain Korea 21 Project, Yonsei University College of Medicine, Seoul, Republic of Korea; 7 Woo Choo Lee Institute for Precision Drug Development, Yonsei University College of Medicine, Seoul, Republic of Korea; 8 Department of Oral Biology, BK21 PLUS Project, Yonsei University College of Dentistry, Seoul, Republic of Korea; 9 Precision Medicine Research Center, College of Medicine, The Catholic University of Korea, Seoul, Republic of Korea; 10 Department of Microbiology, College of Medicine, The Catholic University of Korea, Seoul, Republic of Korea; 11 Cancer Evolution-Ecology Systems Research Center, College of Medicine, The Catholic University of Korea, Seoul, Republic of Korea; 12 Department of Neurosurgery, Brain Tumor Center, Severance Hospital, Yonsei University College of Medicine, Seoul, Republic of Korea; 13 Graduate School of Medical Science, Brain Korea 21 Project, Yonsei University College of Medicine, Seoul, Republic of Korea; 14 Center for Multi-omics, Medical Science Research Institute,, Kyung Hee University College of Medicine, Seoul, Republic of Korea; 15 Department of Pathology, College of Medicine, The Catholic University of Korea, Seoul, Republic of Korea; 16 Department of Bioinspired Science, College of Natural Science, Ewha Womans University, Seoul, Republic of Korea

**Keywords:** CNS cancer, Cancer epigenetics

## Abstract

N-terminal acetyltransferase D (NatD) acetylates histones H4 and H2A at serine 1 and has been implicated in oncogenesis, yet its role in glioblastoma (GBM) remains unexplored. Here, we show that NatD expression is significantly elevated in GBM at both transcript and protein levels. NatD knockdown markedly suppressed proliferation, colony formation, migration, invasion and tumoursphere formation in GBM cell lines and patient-derived models, indicating a critical role in maintaining cancer stem-like properties. These inhibitory effects were rescued by reintroduction of wild-type NatD but not by a catalytically inactive mutant, confirming dependence on its enzymatic activity. Transcriptomic profiling identified fibroblast growth factor receptor (FGFR) 4 as a key downstream effector. Mechanistically, NatD maintained N-terminal acetylation of histones H4 and H2A at the *FGFR4* promoter, thereby antagonizing casein kinase 2α-mediated phosphorylation and sustaining an open chromatin state permissive for transcription. NatD depletion reduced FGFR4 expression and downstream ERK1/2 and AKT phosphorylation, whereas FGFR4 overexpression restored malignant phenotypes suppressed by NatD knockdown both in vitro and in xenograft models. Orthotopic xenograft models further demonstrated that NatD depletion suppresses intracranial tumour growth and prolongs survival, whereas FGFR4 re-expression partially restores tumour progression. Analysis of TCGA GBM datasets revealed that the positive correlation between *NatD* and *FGFR4* expression is maintained across multiple molecular subtypes. Collectively, these findings identify NatD as an oncogenic regulator of GBM and underscore NatD–FGFR4 signalling as a promising therapeutic target.

## NatD Drives Glioblastoma Progression Through FGFR4 Regulation

Glioblastoma (GBM) is a highly aggressive brain tumour with a poor prognosis, necessitating novel therapeutic targets. This study identifies N-terminal acetyltransferase D (NatD) as a key oncogenic driver in GBM, linking its activity to fibroblast growth factor receptor 4 (FGFR4) signalling. Researchers used transcriptomic profiling and functional assays to show that NatD maintains N-terminal acetylation of histones H4 and H2A, promoting *FGFR4* transcription and downstream MAPK/AKT signalling. NatD depletion reduced GBM cell proliferation, migration, invasion, and tumoursphere formation, whereas FGFR4 overexpression restored these malignant traits. In vivo, NatD knockdown suppressed tumour growth and prolonged survival in xenograft models, highlighting the NatD–FGFR4 axis as a potential therapeutic target. These findings suggest that targeting NatD enzymatic activity could inhibit GBM progression, offering a promising direction for future research and therapy development.

This summary was initially drafted using artificial intelligence, then revised and fact-checked by the author.

## Introduction

Glioblastoma (GBM), an IDH-wildtype diffuse astrocytic tumour classified as WHO grade IV, represents the most aggressive and prevalent primary brain tumour in adults^1^. GBM is characterized by rapid proliferation, diffuse infiltration into surrounding brain tissue and a high rate of recurrence^2^. Despite advances in multimodal therapy including surgery, radiotherapy and chemotherapy, the prognosis remains poor, with a median survival of only 12–15 months and recurrence almost inevitable^3,4^. At the molecular level, GBM harbours recurrent genetic alterations such as EGFR amplification, PTEN loss, TERT promoter mutation and combined chromosome 7 gain with chromosome 10 loss, which collectively drive uncontrolled proliferation and therapeutic resistance^1,5,6^. In addition, the tumour microenvironment (TME) is profoundly immunosuppressive and highly heterogeneous, further contributing to treatment failure^7,8^. Despite extensive efforts to delineate its molecular underpinnings, particularly at the level of transcriptional regulation, the fundamental drivers of GBM progression and recurrence remain incompletely understood, highlighting the urgent need to identify novel therapeutic targets.

Increasing evidence indicates that epigenetic dysregulation plays a pivotal role in GBM pathogenesis, with histone modifications emerging as key determinants of tumour progression and therapeutic resistance^9–12^. Diverse histone modifications, including acetylation, methylation and phosphorylation, dynamically regulate chromatin accessibility and transcriptional programs that govern tumour cell survival, maintenance of stem-like properties and therapeutic resistance in GBM. Beyond the effects of individual marks, recent studies have revealed extensive crosstalk among distinct histone modifications, whereby coordinated activities of histone-modifying enzymes reinforce malignant transcriptional states and cellular plasticity in GBM^11,13^. Consistent with this concept, epigenetic regulators such as histone acetyltransferases, deacetylases, methyltransferases and demethylases have gained increasing attention as potential therapeutic targets^14–18^. Although histone deacetylase (HDAC) inhibitors have demonstrated anti-tumour activity in preclinical GBM models, their limited clinical efficacy underscores the need to identify additional, functionally critical epigenetic vulnerabilities that drive GBM aggressiveness and treatment resistance^19^. In this context, elucidating novel epigenetic regulators that integrate oncogenic signalling with chromatin remodelling is essential for understanding GBM progression and for developing more effective therapeutic strategies.

Among histone-modifying enzymes, N-terminal acetyltransferase D (NatD, also known as N-α-acetyltransferase (NAA) 40) specifically acetylates the α-amino group of the N-terminal serine residue (Ser1) of histones H4 and H2A within the conserved SGRG motif^20,21^. Elevated NatD expression has been reported across multiple malignancies, where it promotes tumour growth and invasion through diverse mechanisms^22–24^. For instance, in lung cancer, NatD-mediated H4S1 acetylation antagonizes casein kinase (CK) 2α-dependent phosphorylation and promotes epithelial–mesenchymal transition (EMT) via Slug activation^25^; in breast cancer, NatD enhances invasion through the NatD–FOXA2–MMP14 axis^26^; and in colorectal cancer, NatD supports proliferation and suppresses apoptosis independently of *p53* status^27^. NatD was also proposed as a prognostic marker in aggressive liver cancers^28^.

However, the molecular role of NatD in GBM remains poorly defined. In this study, we sought to elucidate the oncogenic functions of NatD in GBM and to identify its downstream effectors that mediate malignant phenotypes. Our findings demonstrate that NatD promotes tumour progression through epigenetic activation of the fibroblast growth factor receptor (FGFR) 4 signalling pathway, highlighting the NatD–FGFR4 axis as a potential therapeutic vulnerability in GBM.

## Materials and methods

### Analysis of The Cancer Genome Atlas datasets

*NatD* mRNA expression in tumour versus normal tissues was compared using The Cancer Genome Atlas (TCGA) datasets (see Supplementary Methods for details).

### Tissue microarray and immunohistochemistry

Commercial human GBM and normal brain tissue microarrays (TissueArray.Com LLC) were used to evaluate NatD and FGFR4 protein expression. Antibody information is provided in Supplementary Table 1, and the staining protocol is described in Supplementary Methods.

### Cell culture

LN229, U-87 MG and U-373 MG cell lines, as well as patient-derived GBM stem cells, were used in this study (see Supplementary Methods for details). The use of patient-derived GBM tissue was approved by the Institutional Review Board of the National Cancer Center, Korea (NCC2019-0241).

### Lentiviral plasmid construction and stable cell-line generation

Gene knockdown and reconstitution were performed using short hairpin RNAs (shRNAs) and lentiviral vectors (see Supplementary Methods for details).

### Immunoblot analysis

Protein expression was analysed by immunoblot analysis as described in Supplementary Methods.

### Functional assays

Cell proliferation, colony formation, migration and invasion assays were performed as described in Supplementary Methods.

### Extreme limiting dilution assay

GBM stemness was evaluated by extreme limiting dilution assay (ELDA) as described in Supplementary Methods.

### RNA sequencing and bioinformatics

RNA sequencing was performed on LN229 cells stably expressing control shRNA, shRNA targeting *NatD* (shNatD#4), and shNatD#4 rescued with lentivirus expressing wild-type NatD (shNatD/NatD-WT). RNA sequencing data have been deposited in the Gene Expression Omnibus under accession number GSE312750. Analytical details are provided in Supplementary Methods.

### Chromatin immunoprecipitation assay

Chromatin immunoprecipitation (ChIP) assays were conducted using antibodies against histone modifications, pan-histone H3/H4 for normalization or IgG as a negative control. Precipitated DNA was quantified by quantitative real-time PCR (qPCR) with primers targeting the *FGFR4* promoter (see Supplementary Methods for details). Antibody and primer information are listed in Supplementary Tables 1 and 2, respectively.

### Mouse xenograft model

All animal procedures were approved by the Ewha Womans University IACUC (ESM 23-046). BALB/c nude mice (6-week-old males) were injected subcutaneously with 1 × 10^6^ cells. Tumour volume was measured every 3 days and calculated as V = 1/2 × (width)^2^ × length. Mice were sacrificed on day 36, and tumours were harvested for analysis.

### Orthotopic xenograft models

All animal procedures were approved by the Institutional Animal Care and Use Committee of Yonsei University College of Medicine (2023-2027). Orthotopic xenograft models were established by stereotactic intracranial implantation of LN229 cells into the striatum of 6-week-old male BALB/c nude mice. Tumour growth was assessed by MRI, and survival was analysed using the Kaplan–Meier method. Detailed procedures are described in the Supplementary Methods.

### Statistical analysis

All statistical analyses were performed using GraphPad Prism 9. Data are presented as mean ± SEM from at least three independent experiments unless otherwise indicated. Comparisons between two groups were made using Welch’s *t* test, and multiple comparisons were analysed by one-way analysis of variance with Bonferroni correction. A *P* value < 0.05 was considered statistically significant.

## Results

### NatD is upregulated in glioblastoma

**Fig. 1:**
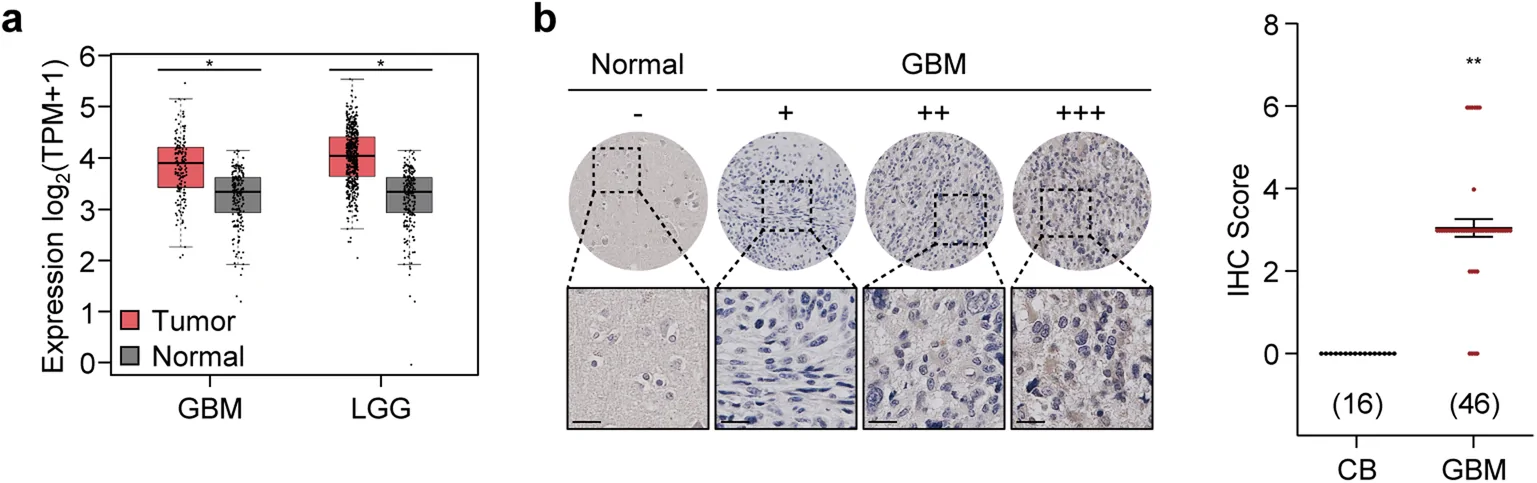
NatD is upregulated in glioblastoma. **a**, The Cancer Genome Atlas RNA sequencing data showing *NatD* mRNA expression in normal brain, low-grade glioma (LGG; tumour *n* = 516, normal *n* = 207), and glioblastoma (GBM) (tumour *n* = 163, normal *n* = 207). Boxplots indicate the median and interquartile range (IQR), with whiskers extending to 1.5 × IQR. **b**, Representative immunohistochemistry (IHC) staining of NatD in normal cerebrum (CB, *n* = 16) and GBM tissues (*n* = 46) with H-score quantification (right). Scale bar, 20 μm. Data are presented as mean ± SEM. Statistical analyses: The Cancer Genome Atlas comparisons (**a**) were assessed using the Wilcoxon rank-sum test; IHC H-scores (**b**) were analysed using the Mann–Whitney *U* test (see Materials and methods, and Supplementary Methods for details). Statistical significance: *P < 0.05, **P < 0.01.

Analysis of TCGA datasets showed that *NatD* mRNA levels were significantly elevated in both low-grade glioma (LGG) and GBM compared with normal brain tissue (Fig. 1). Immunohistochemical analysis of a tissue microarray confirmed higher NatD protein expression in GBM tissues than in normal controls (Fig. 1). These data demonstrate that NatD is overexpressed at both transcript and protein levels in GBM, suggesting its potential oncogenic role.

### NatD depletion suppresses proliferation, colony formation, migration and invasion of glioblastoma cells

**Fig. 2:**
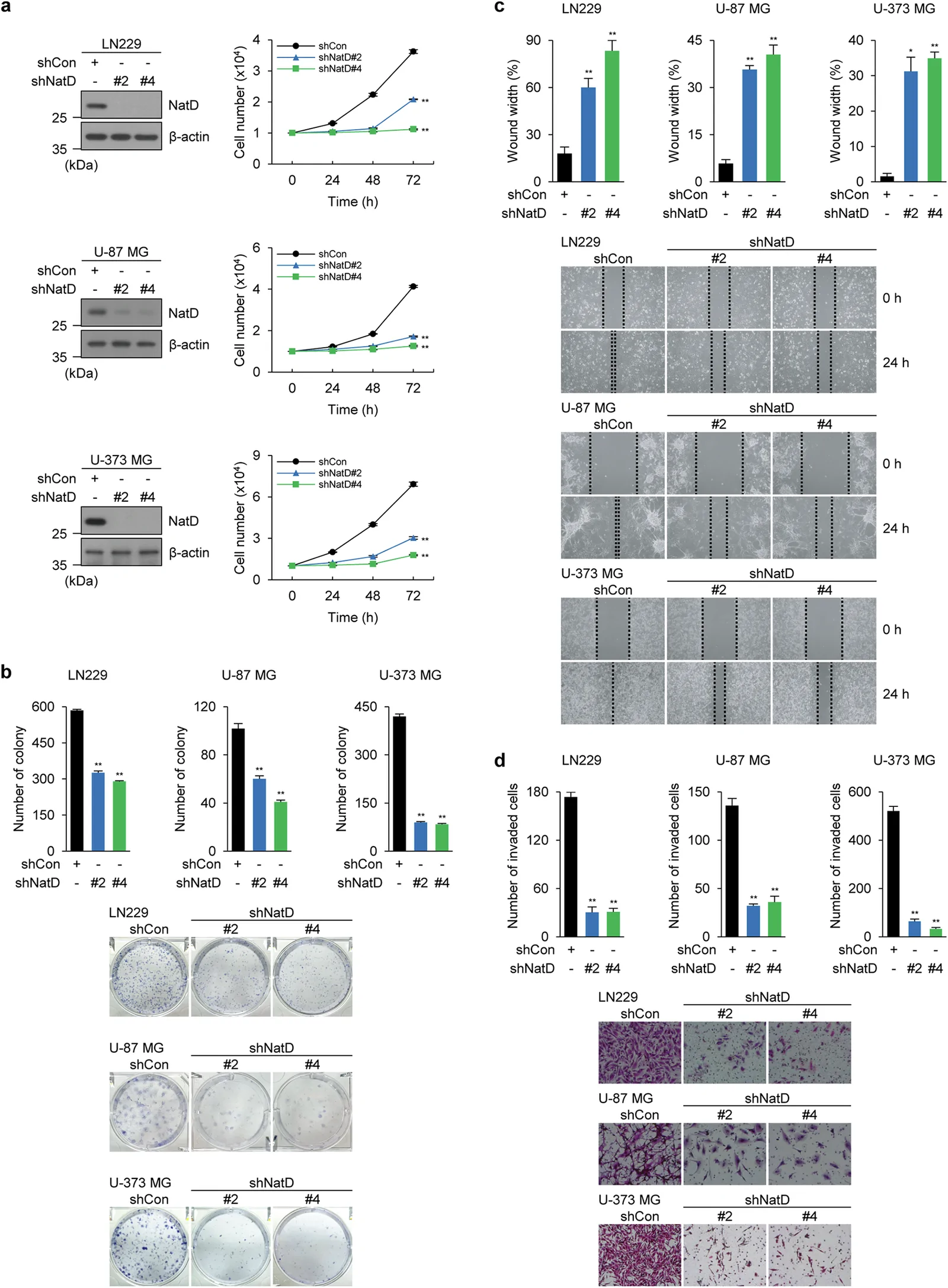
NatD depletion suppresses proliferation, colony formation, migration and invasion of glioblastoma cells. LN229, U-87 MG and U-373 MG cells stably expressing control shRNA (shCon) or one of two independent shRNAs targeting *NatD* (shNatD#2 and shNatD#4) were used in panels **a**–**d**. Representative images (lower) and corresponding quantification (upper) are shown in panels **b**–**d**. **a**, Proliferation assays of glioblastoma (GBM) cells. (Left) Immunoblot validation of stable NatD knockdown in GBM cells. β-actin served as a loading control. (Right) Cells were plated at 10,000 cells per well and counted using a haemocytometer every 24 h for up to 72 h. **b**, Colony formation assays. Cells were plated at 500 cells per well, and colonies were stained and counted on day 14. **c**, Wound-healing assays. Confluent monolayers were scratched, and wound closure was quantified at 24 h. **d**, Transwell invasion assays. Cells were seeded in Matrigel-coated 8-μm pore chambers under serum-free conditions. Invaded cells were fixed, stained and counted after 16 h. Data are presented as mean ± SEM of three independent experiments. Statistical significance was determined using one-way analysis of variance. *P < 0.05, **P < 0.01. Detailed methods are provided in Materials and methods, and Supplementary Methods.

Stable knockdown of NatD in LN229, U-87 MG and U-373 MG cells markedly reduced proliferation and viability (Fig. 2a and Supplementary Fig. 1). Consistent with this, clonogenic potential was significantly decreased upon NatD silencing (Fig. 2b). Wound-healing and Matrigel invasion assays revealed pronounced suppression of cell migration and invasion in NatD-depleted cells (Fig. 2c, d). Together, these findings demonstrate that NatD is essential for maintaining the proliferative and invasive capacities of GBM cells.

### NatD is required for proliferation and self-renewal of glioblastoma stem-like cells

**Fig. 3:**
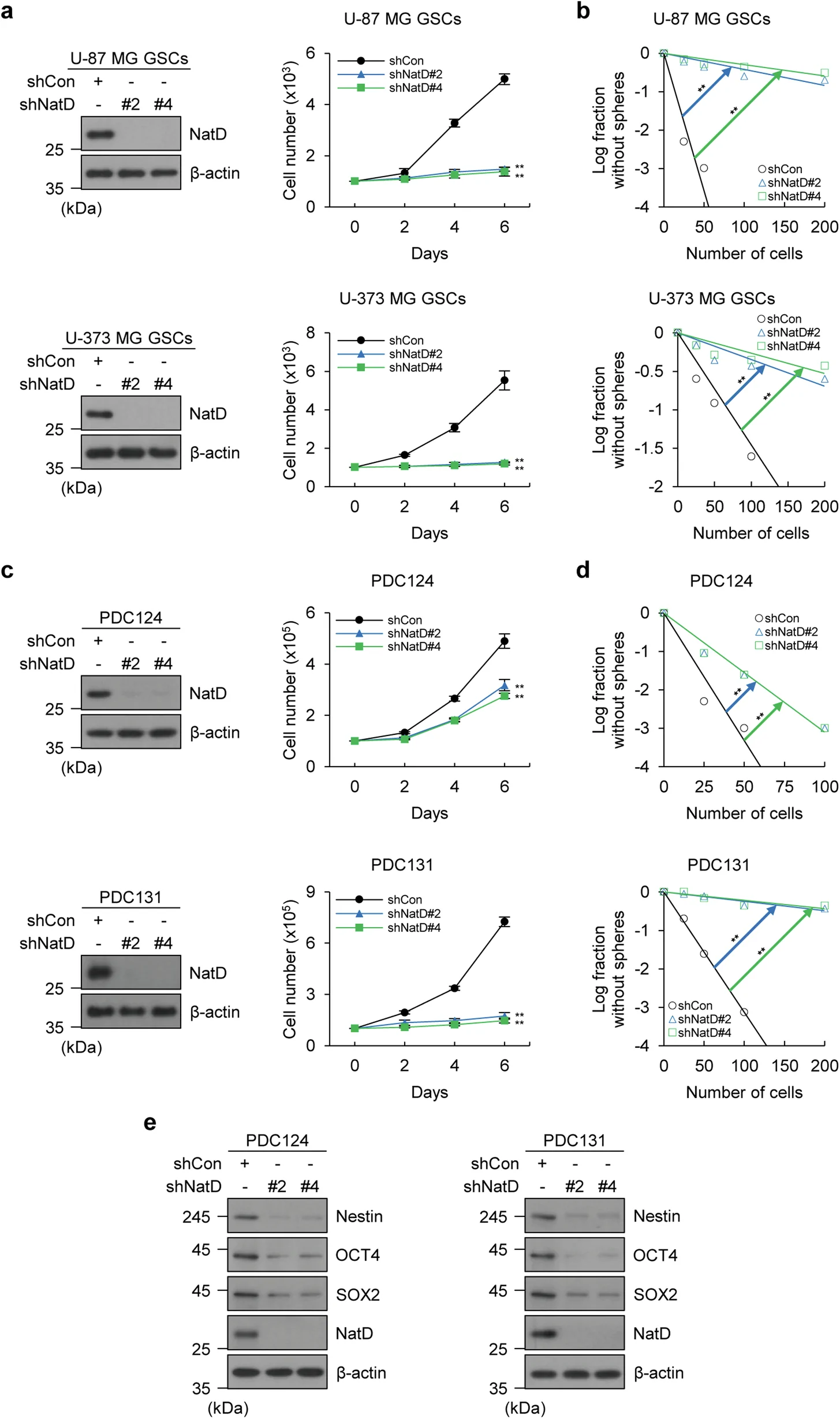
NatD is required for proliferation and self-renewal of glioblastoma stem-like cells. **a** Proliferation assays of glioblastoma stem-like cells (GSCs). (Left) Immunoblot validation of stable NatD knockdown in U-87 MG GSCs and U-373 MG GSCs expressing control shRNA (shCon) or one of two independent shRNAs targeting *NatD* (shNatD#2 and shNatD#4). (Right) GSCs were plated at 1,000 cells per well and counted using a haemocytometer every 48 h for up to 6 days. **b**, Extreme limiting dilution assays (ELDAs). U-87 MG GSCs and U-373 MG GSCs were plated at 200, 100, 50 and 25 cells per well in 96-well ultra-low attachment plates. Tumourspheres were scored on day 14, and stem-cell frequency was calculated using ELDA software. **c**, Proliferation assays of patient-derived glioblastoma stem cells (PDCs). (Left) Immunoblot validation of stable NatD knockdown in PDC124 and PDC131 using two independent short hairpin RNAs (shRNAs) targeting *NatD* (shNatD#2 and shNatD#4) with control shRNA (shCon). (Right) PDCs were plated at 100,000 cells per well and counted every 48 h for up to 6 days. **d**, ELDA in PDCs. PDC124 and PDC131 were assayed as described in **b**, and tumourspheres were scored on day 14. **e**, Immunoblot analysis of PDCs showing expression of indicated stemness-associated proteins. β-actin was used as a loading control in all immunoblots. Data are presented as mean ± SEM of three independent experiments. Statistical significance was determined using one-way analysis of variance. *P < 0.05, **P < 0.01. Detailed methods are provided in Materials and methods, and Supplementary Methods.

Tumoursphere formation assays showed that NatD knockdown significantly reduced both the number and diameter of spheres derived from U-87 MG glioblastoma stem-like cells (GSCs) and U-373 MG GSCs (Supplementary Fig. 2). Correspondingly, proliferation of these GSCs was markedly impaired following NatD silencing (Fig. 3). ELDAs confirmed a decrease in sphere-forming frequency upon NatD knockdown (Fig. 3b). Specifically, stem-cell frequency was reduced by two independent shRNAs, decreasing from 1/13 to 1/194 (shNatD#2) and 1/278 (shNatD#4) in U-87 MG GSCs, and from 1/51 to 1/236 (shNatD#2) and 1/297 (shNatD#4) in U-373 MG GSCs, respectively (Supplementary Table 3). In patient-derived GBM stem cells (PDC124 and PDC131), NatD depletion similarly suppressed proliferation (Fig. 3c). ELDA analysis revealed a marked reduction in stem-cell frequency, with an approximately two-fold and 13-fold decrease following NatD knockdown in PDC124 and PDC131, respectively (Fig. 3d and Supplementary Table 3). Consistent with the ELDA results, NatD depletion reduced the expression of stemness-associated markers, including Nestin, OCT4 and SOX2 (Fig. 3e). Collectively, these results indicate that NatD is required for the maintenance of proliferation and self-renewal capacity in GSCs.

### The oncogenic function of NatD depends on its N-terminal acetyltransferase activity

**Fig. 4:**
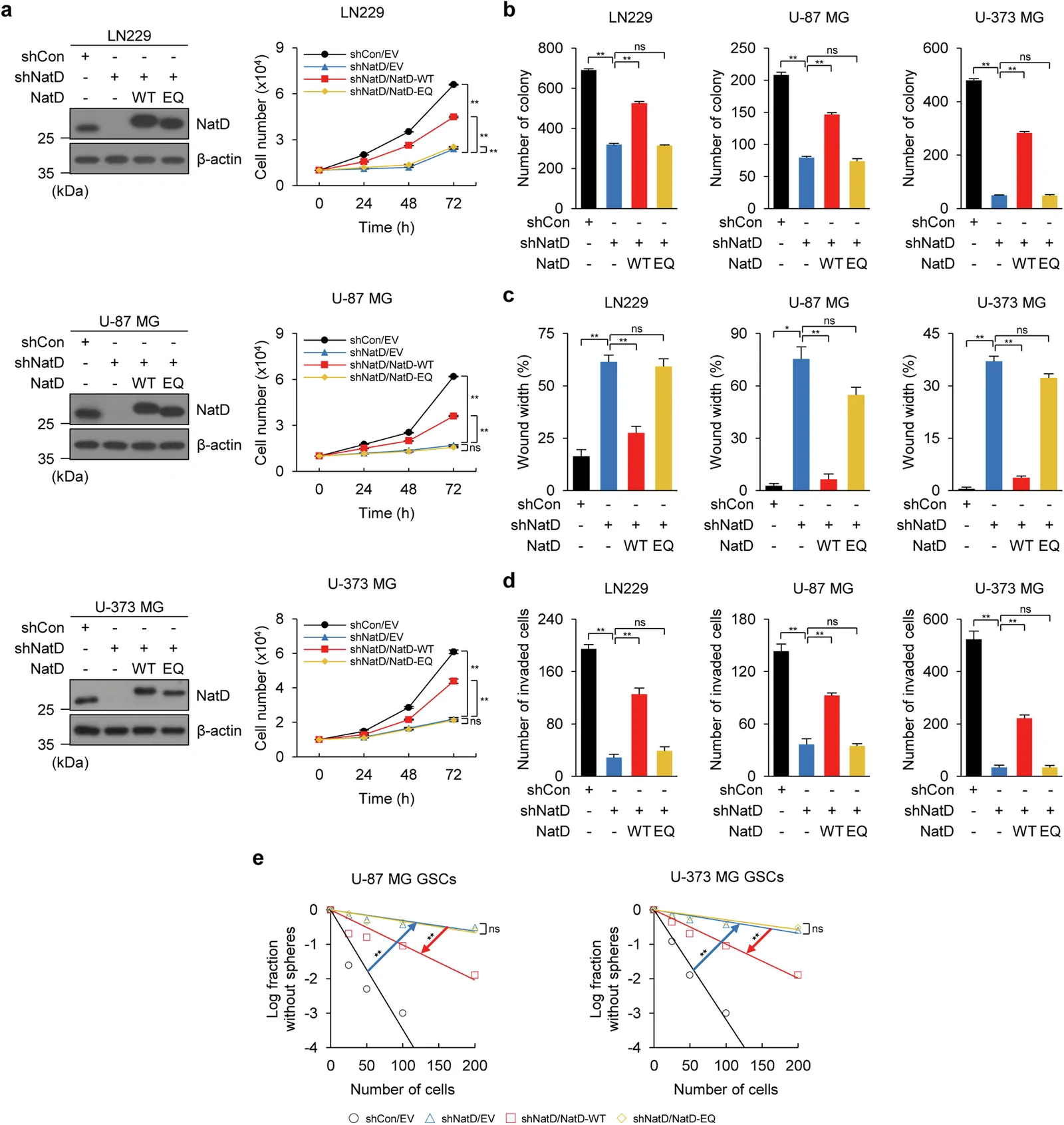
The oncogenic function of NatD depends on its N-terminal acetyltransferase activity. LN229, U-87 MG and U-373 MG cells stably expressing control shRNA (shCon) with empty vector (shCon/EV), shNatD#4 with empty vector (shNatD/EV), shNatD#4 with NatD-WT-FLAG (shNatD/NatD-WT), or shNatD#4 with NatD-EQ-FLAG (shNatD/NatD-EQ) were used in panels **a**–**d**. **a** Immunoblot analysis of NatD expression with β-actin as a loading control (left) and proliferation assays (right). Cells were plated at 10,000 cells per well and counted using a haemocytometer every 24 h for up to 72 h. **b** Colony-formation assays. Cells were plated at 500 cells per well, and colonies were stained and counted on day 14. **c** Wound-healing assays. Confluent monolayers were scratched, and wound closure was quantified at 24 h. **d** Transwell invasion assays. Cells were seeded in Matrigel-coated 8-μm pore chambers under serum-free conditions. Invaded cells were fixed, stained and counted after 16 h. **e** Extreme limiting dilution assays of U-87 MG glioblastoma stem-like cells (GSCs) and U-373 MG GSCs expressing the indicated constructs. Cells were plated at 200, 100, 50 and 25 cells per well in 96-well ultra-low attachment plates. Tumourspheres were scored on day 14, and stem-cell frequency was calculated using extreme limiting dilution assay software. Data are presented as mean ± SEM of three independent experiments. Statistical significance was determined using one-way analysis of variance followed by Tukey’s multiple comparison test. *P < 0.05, **P < 0.01; ns, not significant. Detailed methods are provided in Materials and methods, and Supplementary Methods.

Given that NatD silencing impaired proliferation and self-renewal, we next examined whether its enzymatic activity is required for these effects. The enzymatic deficiency of the catalytically inactive NatD mutant (NatD-E139Q^29^, hereafter NatD-EQ) was independently verified by an in vitro DTNB-based acetylation assay (Supplementary Fig. 3; see Supplementary Methods). Re-expression of wild-type NatD (NatD-WT) but not NatD-EQ, restored proliferation (Fig. 4a), colony formation (Fig. 4b and Supplementary Fig. 4a), migration (Fig. 4c and Supplementary Fig. 4b), and invasion (Fig. 4d and Supplementary Fig. 4c). GSC self-renewal was also restored by re-expression of NatD-WT with stem-cell frequency increased from 1/263 to 1/77 in U-87 MG GSCs and from 1/247 to 1/89 in U-373 MG GSCs (Fig. 4e and Supplementary Table 3). These data demonstrate that the N-terminal acetyltransferase activity of NatD is indispensable for its oncogenic role in GBM.

### *FGFR4* is transcriptionally regulated by NatD enzymatic activity

**Fig. 5:**
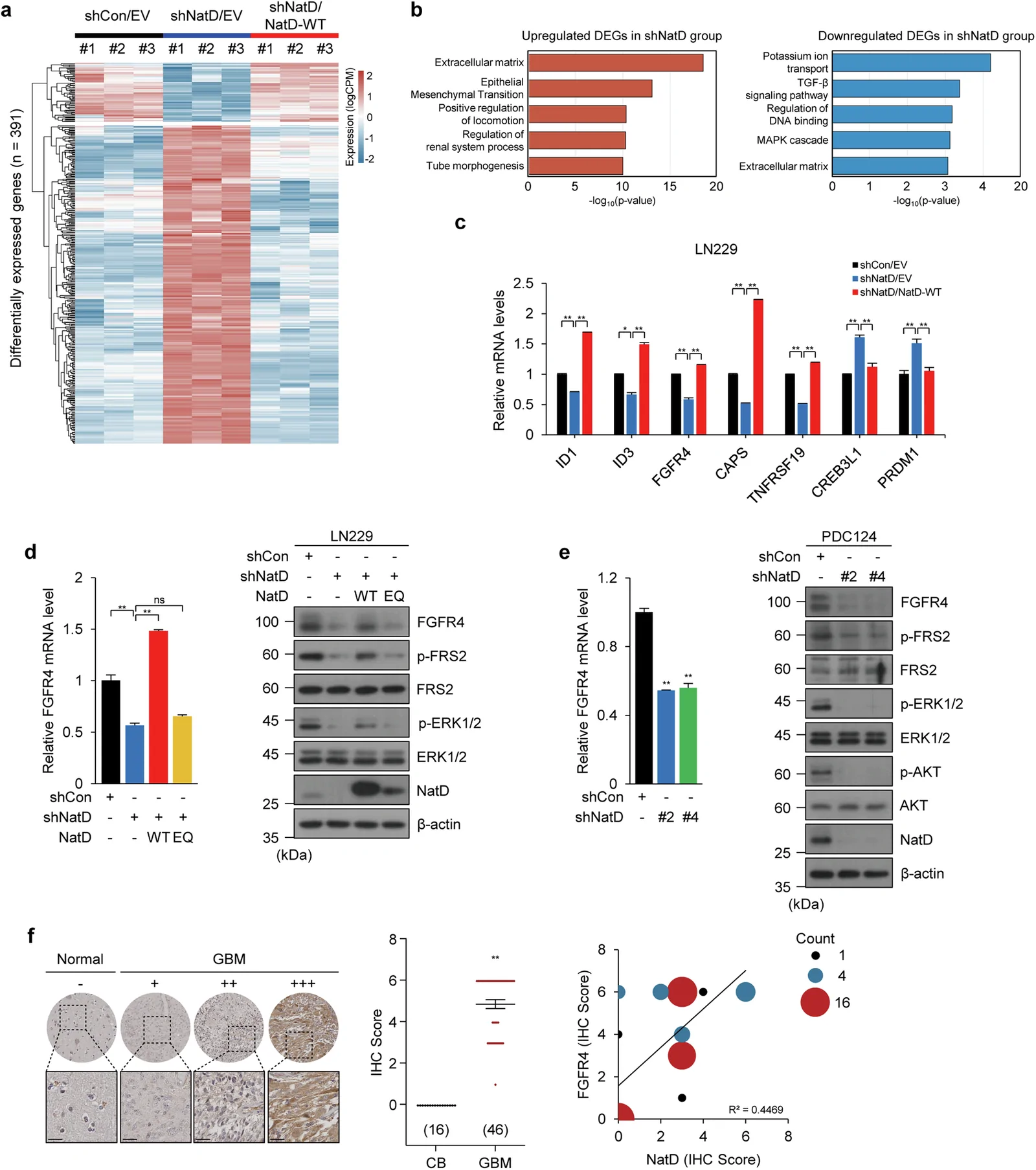
FGFR4 is transcriptionally regulated by NatD enzymatic activity. **a** A heatmap of differentially expressed genes (DEGs) identified by RNA sequencing in LN229 cells stably expressing control shRNA (shCon) with empty vector (shCon/EV), shNatD#4 with empty vector (shNatD/EV) and shNatD#4 with NatD-WT-FLAG (shNatD/NatD-WT). **b** Pathway enrichment analysis showing representative pathways upregulated (left) and downregulated (right) upon NatD depletion. **c** Quantitative real-time RT-PCR (qRT-PCR) validation of selected DEGs, including *FGFR4*, in LN229 cells. **d** LN229 cells stably expressing control shRNA (shCon) with empty vector, shNatD#4 (shNatD) with empty vector and shNatD#4 with lentiviral constructs (NatD-WT or NatD-EQ) were subjected to qRT-PCR analysis of *FGFR4* (left) and immunoblot analysis of the indicated proteins (right). **e** Patient-derived glioblastoma stem cells (PDC124) expressing control shRNA (shCon) or shNatD (#2, #4) were subjected to qRT-PCR (left) and immunoblot analysis of the indicated proteins (right). **f** Representative immunohistochemistry (IHC) images (left) of FGFR4 in normal cerebrum (CB, *n* = 16) and GBM tissues (*n* = 46) from the same tissue microarray cohort, with H-score quantification (middle). Scale bar, 20 μm. Correlation analysis between FGFR4 and NatD expression in the same cohort (right). β-actin was used as an internal control for qRT-PCR and as a loading control for immunoblot analyses. Data are presented as mean ± SEM of three independent experiments. Statistical significance was determined using one-way analysis of variance followed by Tukey’s multiple comparison test. *P < 0.05, **P < 0.01; ns, not significant. Detailed methods are provided in Materials and methods, and Supplementary Methods.

To identify downstream effectors mediating NatD function, RNA sequencing analysis was performed in LN229 cells. NatD knockdown resulted in 757 upregulated and 234 downregulated genes, of which 391 genes were restored by NatD reconstitution (Fig. 5a and Supplementary Tables 4, 5). Pathway enrichment of these 391 genes highlighted epithelial–mesenchymal transition (*P* = 8.0 × 10^−14^), TGFβ signalling (*P* = 6.4 × 10^−5^), MAPK cascade (*P* = 6.5 × 10^−4^) and potassium ion transport (*P* = 4.2 × 10^−4^) (Fig. 5b and Supplementary Table 6). Given that both MAPK and TGFβ signalling pathways were attenuated upon NatD knockdown, *FGFR4*, which encodes a receptor tyrosine kinase linked to MAPK, PI3K/AKT and TGFβ pathways implicated in GBM progression^30–33^, emerged as a prime candidate. Quantitative real-time RT-PCR (qRT-PCR) confirmed that NatD depletion reduced *FGFR4* mRNA expression, along with several additional candidates including *inhibitor of DNA binding* (*ID*) *1*, *ID3* and *TNF receptor superfamily* (*TNFRSF*) *19* (Fig. 5c). Importantly, re-expression of NatD-WT, but not the catalytic mutant NatD-EQ, restored *FGFR4* mRNA levels (Fig. 5d and Supplementary Fig. 5a), supporting a transcriptional mechanism dependent on NatD enzymatic activity. Consistently, immunoblotting demonstrated decreased FGFR4 protein levels and reduced phosphorylation of its downstream effectors fibroblast growth factor receptor substrate 2 (FRS2), ERK1/2 and AKT upon NatD depletion; these effects were rescued by re-expression of NatD-WT but not the catalytic mutant NatD-EQ (Fig. 5d and Supplementary Fig. 5b). This reduction in FGFR4 expression and downstream signalling was further recapitulated in patient-derived GBM stem cells (PDC124 and PDC131 cells, Fig. 5e and Supplementary Fig. 5c). Moreover, immunohistochemical analysis of clinical GBM specimens revealed a moderate positive correlation between NatD and FGFR4 expression (R^2^ = 0.4469, Fig. 5f), supporting the potential clinical relevance of the NatD–FGFR4 axis. Collectively, these findings identify *FGFR4* as a transcriptional target of NatD and a key mediator of NatD-driven oncogenic signalling in GBM.

### NatD regulates *FGFR4* transcription via histone modifications

**Fig. 6:**
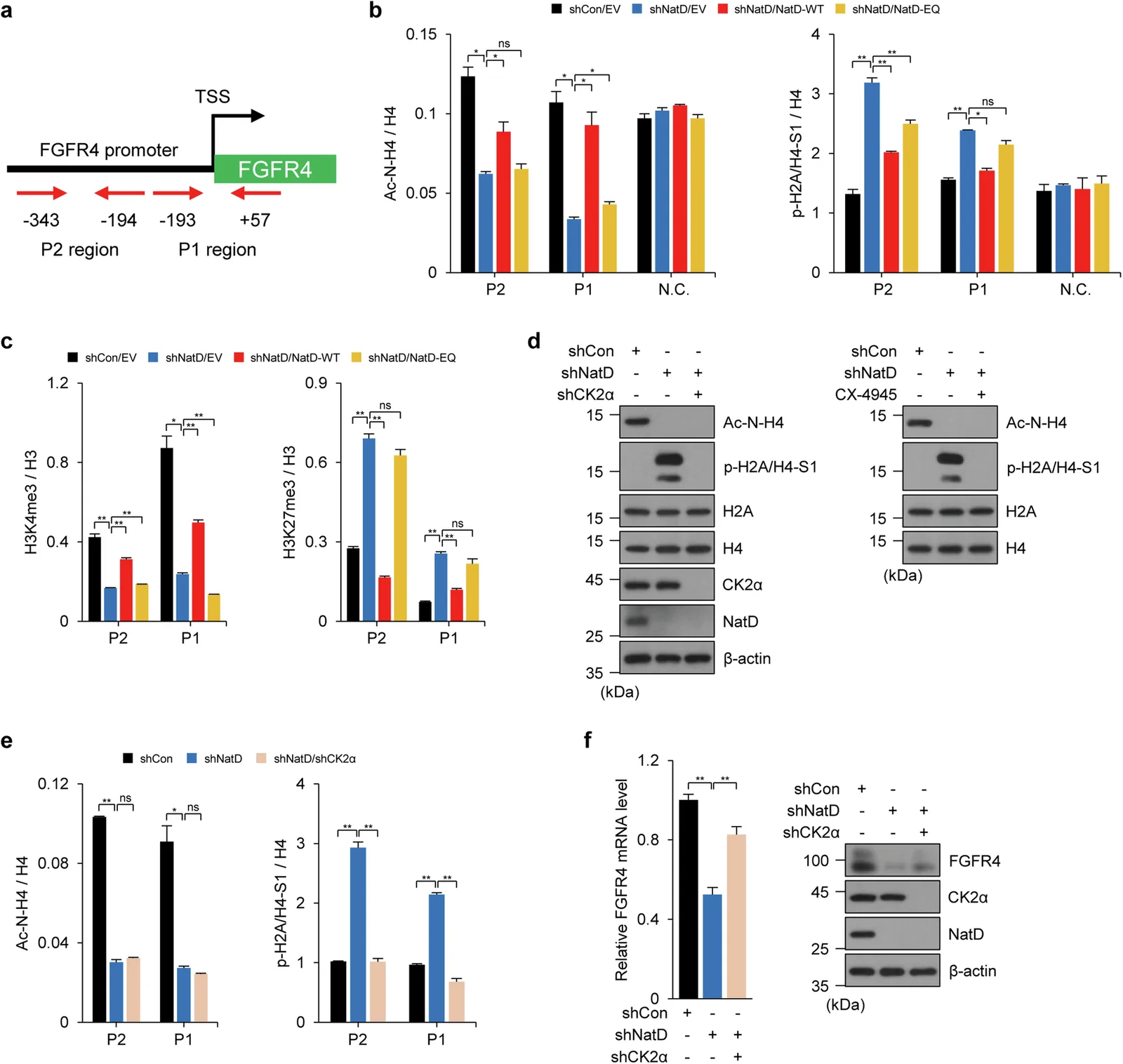
NatD regulates *FGFR4* transcription through histone modifications. **a** Schematic representation of the *FGFR4* promoter showing the two promoter-proximal chromatin immunoprecipitation (ChIP)-quantitative PCR (qPCR) amplicons (P2: –343 to –194; P1: –193 to +57 relative to the transcription start site (TSS)). **b** ChIP-qPCR analysis of LN229 cells stably expressing control shRNA with empty vector (shCon/EV), shNatD#4 with empty vector (shNatD/EV), shNatD#4 with NatD-WT-FLAG (shNatD/NatD-WT) and shNatD#4 with NatD-EQ-FLAG (shNatD/NatD-EQ) using antibodies against N-terminal acetylated H4 (Ac-N-H4) and phosphorylated H2A/H4 at serine 1 (p-H2A/H4-S1). Pan-H4 (total H4) ChIP was used for normalization and IgG served as a negative control. N.C. denotes a distal negative-control region located ~20 kb downstream of the *FGFR4* gene, predicted as a gene-independent site by UniBind, showing baseline signal. **c** ChIP-qPCR analysis of H3K4 trimethylation (H3K4me3) and H3K27 trimethylation (H3K27me3) at P2 and P1 under the same experimental conditions as in **b**. Pan-H3 (total H3) ChIP was used for normalization and IgG served as a negative control. **d** LN229 cells stably expressing control shRNA (shCon), shNatD#4 (shNatD) or shNatD#4 with shCK2α were subjected to immunoblot analyses of the indicated proteins (left). LN229 cells stably expressing control shRNA (shCon) or shNatD#4 (shNatD) were treated with vehicle or CX-4945 (20 μM) for 24 h and subjected to immunoblot analysis, with H2A and H4 serving as normalization controls (right). **e** ChIP-qPCR analysis of Ac-N-H4 and p-H2A/H4-S1 at P2 and P1 in LN229 cells expressing the indicated shRNAs as in panel **d**. Pan-H4 (total H4) ChIP was used for normalization and IgG served as a negative control. **f** LN229 cells expressing the indicated shRNAs as in panel **d** were subjected to qRT-PCR for *FGFR4* (left) and immunoblot analysis of indicated proteins (right). β-actin was used as a loading control for immunoblot analyses shown in panels **d** and **f**. Data are presented as mean ± SEM of three independent experiments. Statistical significance was determined using one-way analysis of variance followed by Tukey’s multiple comparison test. *P < 0.05, **P < 0.01; ns, not significant. Detailed methods are provided in Materials and methods, and Supplementary Methods.

ChIP analysis revealed that NatD depletion reduced N-terminal acetylation of histone H4 (Ac-N-H4), while concomitantly increasing phosphorylation of histones H2A and H4 at serine 1 (p-H2A/H4-S1), a modification previously attributed to CK2α activity^25,34^ (Fig. 6b) (the specificity of the custom anti-Ac-N-H4 antibody was validated using peptide-based assays; Supplementary Methods and Supplementary Fig. 6). These changes were accompanied by decreased enrichment of the active chromatin mark H3K4me3 (trimethylated H3K4) and increased enrichment of the repressive mark H3K27me3 (trimethylated H3K27) at the *FGFR4* promoter (Fig. 6c). Notably, these chromatin alterations were reversed by re-expression of wild type NatD (NatD-WT) but not by the catalytically inactive mutant NatD-EQ. Furthermore, genetic knockdown (shCK2α) or pharmacological inhibition (CX-4945) of CK2α abolished the NatD depletion-induced increase in H2A/H4 serine 1 phosphorylation at the *FGFR4* promoter and concomitantly restored FGFR4 expression at both the mRNA and protein levels (Fig. 6d–f). Together, these results indicate that NatD promotes *FGFR4* transcription by sustaining N-terminal acetylation of histones H2A and H4, and antagonizing CK2α-mediated serine 1 phosphorylation, thereby maintaining an open chromatin configuration permissive for transcription.

### FGFR4 mediates the oncogenic function of NatD

**Fig. 7:**
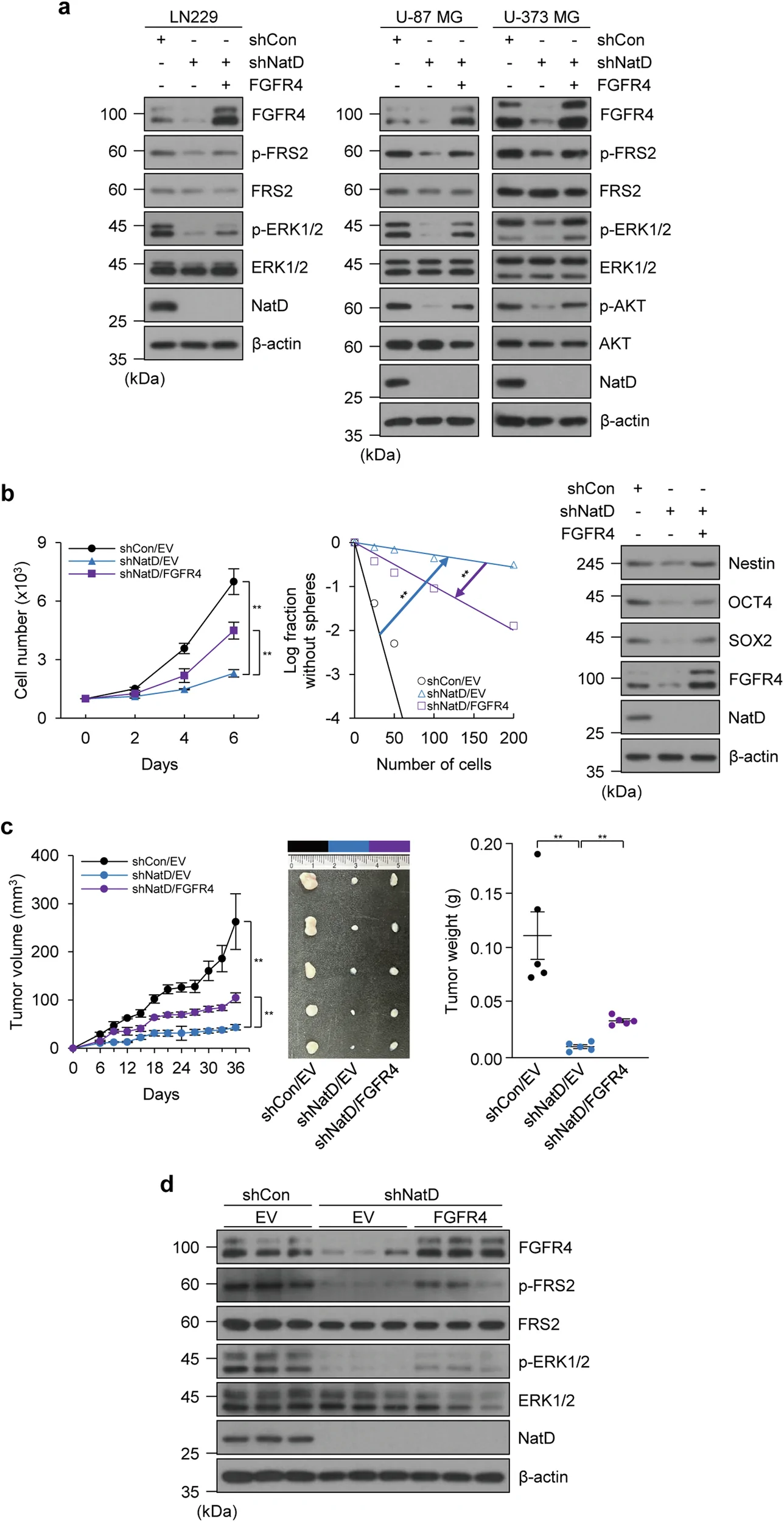
FGFR4 mediates the oncogenic functions of NatD. **a** Immunoblot analysis of glioblastoma cells stably expressing control shRNA (shCon) with empty vector, shNatD#4 (shNatD) with empty vector or shNatD#4 with FLAG-FGFR4 (FGFR4). **b** U-87 MG GSCs expressing control shRNA with empty vector (shCon/EV), shNatD#4 with empty vector (shNatD/EV) or shNatD#4 with FLAG-FGFR4 (shNatD/FGFR4) were subjected to proliferation assays (left), extreme limiting dilution assays (ELDA; middle), and immunoblot analysis of stemness-associated markers (right). **c** LN229 xenograft models demonstrating that FGFR4 re-expression (shNatD/FGFR4) partially rescues tumour growth following NatD knockdown (shNatD/EV). Tumour progression is shown by tumour volume over time (left), representative tumour images (middle), and tumour weight at endpoint (right). **d** Immunoblot analysis of LN229 xenograft tumour tissues showing that FGFR4 re-expression (shNatD/FGFR4) reverses the reduced phosphorylation of FRS2 and ERK1/2 in NatD-depleted (shNatD/EV) tumours. β-actin was used as a loading control for immunoblot analyses shown in panels **a,** **b**, and **d**. Data are presented as mean ± SEM. Statistical significance was determined using one-way ANOVA followed by Tukey’s multiple comparison test. **P < 0.01. Detailed methods are provided in Materials and methods, and Supplementary Methods.

We next assessed whether enforced FGFR4 expression could rescue the oncogenic phenotypes suppressed by NatD depletion. Ectopic expression of FGFR4 in NatD-depleted cells restored cell proliferation, colony formation, migration, invasion and phosphorylation of FRS2, ERK1/2 and AKT (Supplementary Fig. 7 and Fig. 7a). FGFR4 overexpression also rescued GSC proliferation and self-renewal and re-induced the expression of stemness-associated markers (Fig. 7b). Consistent with these findings, the stem-cell frequency, which was reduced from 1/23 to 1/326 upon NatD depletion, was partially restored to 1/86 by FGFR4 overexpression (Supplementary Table 3). In xenograft models, NatD knockdown significantly suppressed tumour growth, which was partially rescued by FGFR4 re-expression (Fig. 7c). Accordingly, FGFR4 restored phosphorylation of FRS2 and ERK1/2 in xenograft tumour tissues (Fig. 7d). Collectively, these results establish FGFR4 as a critical mediator of NatD-driven oncogenicity in GBM.

### The NatD–FGFR4 axis regulates glioblastoma progression in an orthotopic xenograft model

**Fig. 8:**
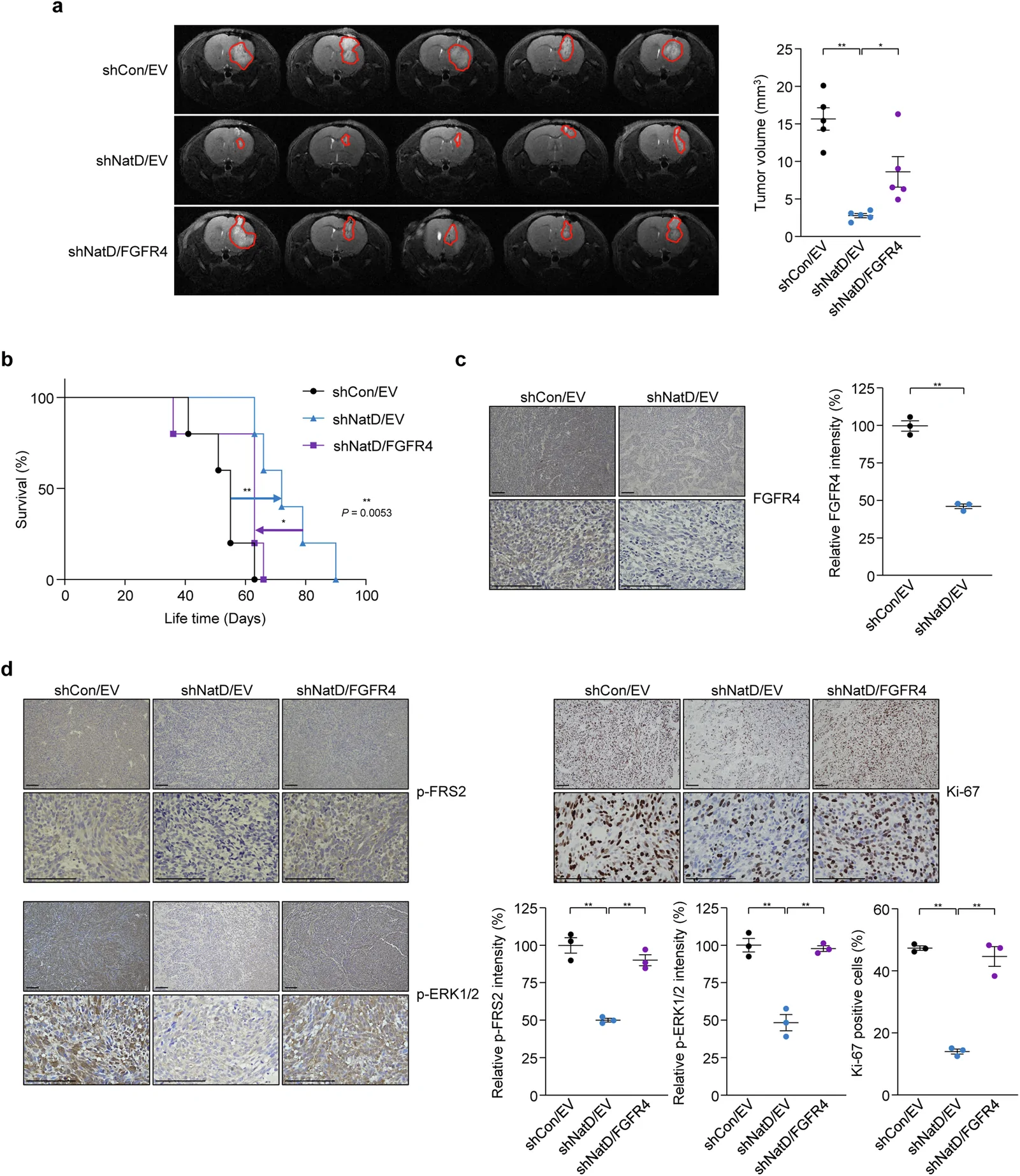
FGFR4 restores tumour growth and survival impaired by NatD depletion in an orthotopic xenograft model. LN229 cells stably expressing control shRNA with empty vector (shCon/EV), shNatD#4 with empty vector (shNatD/EV) or shNatD#4 with FLAG-FGFR4 (shNatD/FGFR4) were used to establish an orthotopic xenograft model. **a** MRI of intracranial tumours at 5 weeks post-implantation. Representative images (left) and quantification of tumour volume (right) are shown. Tumour volumes were calculated using 3D Slicer software (*n* = 5 per group). **b** Kaplan–Meier survival curves of mice bearing orthotopic tumours (*n* = 5 per group). **c**, **d** Representative immunohistochemistry images and corresponding quantification of intracranial tumour sections. **c**, FGFR4. **d** Phosphorylated FRS2 (p-FRS2), phosphorylated ERK1/2 (p-ERK1/2) and Ki-67. Quantification was performed by measuring staining intensity of p-FRS2 and p-ERK1/2, and the percentage of Ki-67-positive cells (*n* = 3 per group). Scale bar, 25 μm. Data are presented as mean ± SEM. Statistical significance was determined using one-way ANOVA followed by Tukey’s multiple comparison test (**a**, **c**, **d**) and the log-rank (Mantel–Cox) test (**b**). *P < 0.05, **P < 0.01. Detailed methods are provided in Materials and methods, and Supplementary Methods.

To evaluate the physiological relevance of the NatD–FGFR4 axis in a brain microenvironment, orthotopic xenograft models were established using LN229 cells expressing control shRNA with empty vector (shCon/EV), *NatD*-targeting shRNA with empty vector (shNatD/EV), or shNatD with FLAG-FGFR4 (shNatD/FGFR4). MRI analysis at 5 weeks post-implantation revealed that NatD depletion markedly suppressed intracranial tumour growth, whereas re-expression of FGFR4 partially restored tumour progression (Fig. 8a). Consistently, quantification of tumour volume demonstrated a significant reduction in tumour burden in the shNatD/EV group compared with the shCon/EV group, which was partially rescued by FGFR4 re-expression. Kaplan–Meier survival analysis demonstrated that NatD depletion significantly prolonged survival, whereas FGFR4 re-expression attenuated this effect (Fig. 8b), indicating that the NatD–FGFR4 axis critically regulates tumour progression in vivo. Immunohistochemical analyses of intracranial tumour tissues further confirmed that FGFR4 re-expression restores FGFR4 signalling and downstream pathway activation, as demonstrated by increased phosphorylation of FRS2 and ERK1/2, along with enhanced proliferative activity indicated by Ki-67 staining (Fig. 8c, d). Collectively, these results demonstrate that the NatD–FGFR4 axis plays a critical role in GBM progression within the brain microenvironment.

## Discussion

This study identifies NatD as a previously unrecognized oncogenic regulator in GBM and elucidates a key mechanistic axis underlying NatD-mediated tumour progression. We demonstrate that NatD is significantly upregulated in GBM and that its enzymatic activity is indispensable for sustaining proliferation, migration, invasion and self-renewal of GSCs. The observation that NatD expression is elevated not only in GBM but also in LGG suggests that NatD activation may represent an early event during gliomagenesis. Although NatD expression is not significantly higher in GBM than in LGG, our findings indicate that NatD enzymatic activity plays a central role in sustaining malignant phenotypes in GBM. Through transcriptomic and functional analyses, we establish FGFR4 as a major downstream effector of NatD, thereby linking NatD-mediated histone modifications to activation of the MAPK and PI3K/AKT signalling pathways. Mechanistically, NatD catalyses the N-terminal acetylation of histones H4 and H2A, thus reshaping local chromatin architecture and promoting *FGFR4* gene transcription. Upregulation of FGFR4, in turn, activates downstream MAPK and PI3K/AKT signalling pathways, supporting oncogenic and stem-like phenotypes in GSCs. Notably, NatD knockdown attenuated the anti-proliferative effect of FGFR4 inhibitor, BLU-554 (Supplementary Fig. 8), consistent with reduced FGFR4 expression in NatD-deficient cells. This finding further supports a functional link between NatD activity and FGFR4 signalling in GBM.

Previous studies have implicated N-terminal acetyltransferases in diverse aspects of tumour biology, including regulation of protein stability, chromatin dynamics and cell signalling^20,21,35^. However, the role of NatD in GBM has remained largely unexplored. Our findings advance this field by demonstrating that NatD directly modulates transcriptional programs through histone acetylation, thereby controlling FGFR4 expression in GBM. FGFR4 activation engages the MAPK and PI3K/AKT cascades, which critically regulate GBM growth, mesenchymal transition and maintenance of stem-like phenotypes^2,36–40^. Consistent with these reports, enforced FGFR4 expression restored ERK1/2 and AKT activation as well as GSC phenotypes suppressed by NatD depletion. In parallel, NatD depletion reduced the expression of stemness-associated transcription factors, including Nestin, OCT4 and SOX2, and impaired tumoursphere formation. Collectively, these findings show a unique epigenetic mechanism by which NatD regulates GBM stemness through the NatD–FGFR4 axis and support a central role for the NatD–FGFR4–MAPK/AKT signalling axis in maintaining GBM oncogenicity.

Transcriptomic analyses further revealed downregulation of TGFβ-related pathway signatures upon NatD depletion. Given that TGFβ signalling is a critical regulator of GSC stemness and that FGFR4 has been reported to modulate TGFβ/SMAD signalling in a context-dependent manner, particularly in fibrotic settings^41–44^, a potential link between the NatD–FGFR4 axis and TGFβ signalling was considered. However, canonical TGFβ target genes, including *ID1* and *ID3*, were not restored by FGFR4 re-expression (Supplementary Fig. 9), suggesting that attenuation of TGFβ-associated transcriptional programs following NatD depletion may occur independently of FGFR4 downregulation. This raises the possibility that NatD could modulate TGFβ signalling through FGFR4-independent mechanisms. For example, NatD-mediated N-terminal acetylation of histones could directly influence chromatin accessibility at TGFβ target gene loci, altering their transcriptional responses. Alternatively, NatD may regulate other chromatin-associated factors or transcriptional co-regulators that impact TGFβ signalling.

Given the well-established role of TGFβ in immune suppression and TME remodelling in GBM, NatD-dependent modulation of TGFβ signalling may have broader implications beyond tumour cell-intrinsic phenotypes, potentially influencing immune surveillance and therapeutic responsiveness^45–47^. In particular, as TGFβ signalling can dampen antitumour immune responses and limit the effectiveness of immunotherapies^48,49^, NatD inhibition could potentially enhance the efficacy of immune checkpoint blockade or other immunotherapeutic strategies by reducing TGFβ-mediated immunosuppression within the TME. Additionally, targeting NatD may disrupt pro-tumorigenic aspects of TGFβ signalling that contribute to resistance against conventional therapies, further broadening the scope of clinical benefit. Further studies will be required to delineate the FGFR4-independent mechanisms by which NatD impacts TGFβ-related pathways in GBM and TME.

Although FGFR4 has been associated with poor prognosis in GBM^32,33^, our study identifies NatD as a critical upstream regulator of FGFR4, introducing a new layer of therapeutic relevance. Unlike broad-spectrum histone acetyltransferases such as CBP/p300 or GCN5, NatD displays unique substrate specificity towards histones H2A/H4 serine 1, linking this modification to direct transcriptional control of *FGFR4* and its oncogenic signalling. From a clinical perspective, NatD represents a promising therapeutic target. Inhibition of its enzymatic activity may suppress GBM growth and stem-cell maintenance, particularly in tumours with elevated FGFR4 signalling. The positive correlation between NatD and FGFR4 expression in patient samples supports this translational potential. Moreover, as several FGFR4 inhibitors are already in clinical use or development^50,51^, our findings raise the possibility that NatD expression level may serve as a potential biomarker for stratifying GBM patients who are more likely to respond to FGFR4-targeted therapies.

In this study, we extended our in vivo analyses by employing an orthotopic xenograft model, which more closely recapitulates the brain microenvironment compared with subcutaneous models. Our results demonstrate that NatD depletion significantly suppresses intracranial tumour growth and prolongs survival, whereas FGFR4 re-expression partially restores tumour progression (Fig. 8). These findings provide strong evidence that the NatD–FGFR4 axis plays a critical role in GBM progression within a physiologically relevant brain context.

Given the molecular heterogeneity of GBM, the NatD–FGFR4 axis may exhibit subtype-specific relevance. Notably, FGFR4-driven MAPK/AKT signalling is consistent with features of the classical type, whereas the enrichment of TGFβ and EMT-related pathways suggests a potential role in the mesenchymal subtype, which is characterized by enhanced invasiveness and inflammatory signalling^46,52^. However, subtype-stratified analysis of TCGA GBM datasets revealed that NatD expression correlates positively with FGFR4 expression across all major molecular subtypes (Supplementary Fig. 10), suggesting that this regulatory axis is not restricted to a specific subtype but represents a broadly relevant mechanism in GBM.

Despite these advances, our study has certain limitations. Although NatD-dependent acetylation of histones H4 and H2A antagonizes CK2α-mediated serine 1 phosphorylation, the precise molecular mechanisms underlying this crosstalk remain to be fully elucidated. Notably, inhibition of CK2α alone was sufficient to restore FGFR4 expression following NatD depletion, suggesting that CK2α-dependent H4/H2A serine 1 phosphorylation represents a dominant repressive mechanism at the *FGFR4* promoter that can override the loss of NatD-mediated N-terminal acetylation. Accordingly, CK2α inhibition may bypass the requirement for NatD-mediated N-terminal acetylation by relieving a dominant inhibitory constraint on *FGFR4* transcription (Fig. 6d, f). In addition, the rescue experiments in this study were performed using ectopic expression systems, which may result in expression levels exceeding those of endogenous NatD or FGFR4. Although the differential effects observed between NatD-WT and the catalytically inactive NatD-EQ mutant strongly support an activity-dependent mechanism, future studies employing approaches that more closely recapitulate endogenous gene regulation, such as CRISPR/dCas9-based gene activation systems, may provide additional physiological insight into the role of NatD in GBM. Furthermore, our in vivo analyses were limited to subcutaneous and orthotopic xenograft models. Although orthotopic xenograft models capture key aspects of tumour growth in the brain, they do not fully reflect the genetic and phenotypic heterogeneity of human GBM. In this regard, patient-derived xenograft (PDX) models may provide a more clinically relevant platform for evaluating tumour behaviour and therapeutic response. Future studies using PDX systems will be important to further validate the role of the NatD–FGFR4 axis and assess its translational potential in heterogeneous GBM settings.

From a therapeutic perspective, effective targeting of the NatD–FGFR4 axis in GBM will require the development of selective NatD inhibitors with central nervous system-penetrant pharmacokinetic properties. The blood–brain barrier represents a major obstacle for the delivery of many small-molecule inhibitors, potentially limiting their efficacy in intracranial tumours^53^. Although potent bisubstrate inhibitors of NatD have been developed, including CoA-peptide-based compounds with high enzymatic potency and selectivity, these molecules remain primarily biochemical or probe-level inhibitors and may have limited drug-like properties for brain delivery^54^. More recently, cell-potent NatD bisubstrate inhibitors have been reported, suggesting progress towards cellular target engagement; however, further optimization will be required to improve blood–brain barrier permeability, adequate brain exposure, metabolic stability and overall pharmacokinetic profiles suitable for intracranial tumours^55^. Thus, future studies should focus on developing central nervous system-penetrant NatD inhibitors or optimized delivery strategies to translate NatD targeting into GBM therapy.

In conclusion, NatD functions as a key epigenetic driver of GBM progression by promoting *FGFR4* transcription and subsequent MAPK/AKT activation, thereby sustaining malignant and stem-like phenotypes. Collectively, these results position the NatD–FGFR4 axis as a promising therapeutic target in GBM.

## Supplementary information

Supplementary Information

Supplementary Table 4

Supplementary Table 5

Supplementary Table 6
