# Supplementary material for: NatD promotes glioblastoma progression through epigenetic activation of FGFR4 signalling
Source: Exp Mol Med. 2026 Sep 2;58(9):2938–50. doi: 10.1038/s12276-026-01818-9 (PMC13633273; doi:10.1038/s12276-026-01818-9)
Supplement: Supplementary file 1 [file 12276_2026_1818_MOESM1_ESM.pdf]

Supplementary Methods

TCGA data analysis

RNA sequencing expression profiles and clinical information for glioblastoma (GBM) and low-grade glioma (LGG) were obtained from The Cancer Genome Atlas (TCGA) via the Genomic Data Commons (GDC) portal. Tumor-versus-normal expression analyses were performed using GEPIA2, which integrates TCGA tumor data with normal tissue data form the Genotype-Tissue Expression (GTEx) project. The GBM dataset comprised 163 tumor and 207 normal samples, while the LGG dataset included 518 tumor and 207 normal samples. For subtype-stratified correlation analysis, TCGA–GBM tumor samples with available RNA sequencing expression data and molecular subtype annotation were analyzed (n = 251). Expression values were normalized as $\log_2(\mathrm{TPM} + 1)$. Comparisons between tumor and normal tissues were performed using the Wilcoxon rank-sum test. Boxplots were generated using GraphPad Prism 9.

Immunohistochemistry (IHC) protocol

Human glioma tissue microarray slides (GL2082a, TissueArray.Com LLC), prepared from formalin-fixed, paraffin-embedded (FFPE) tissues, consisting of 208 tissue cores (1 mm diameter, 5 μm thickness) derived from 104 cases, including GBM (n = 46) and normal cerebrum (CB) tissues (n = 16), were deparaffinized in xylene and rehydrated through graded ethanol. Tumor tissues derived from mouse orthotopic xenograft models were also processed as formalin-fixed, paraffin-embedded (FFPE) sections and subjected to the same immunohistochemical staining procedure. Antigen retrieval was performed in citrate buffer (10 mM sodium citrate, pH 6.0) at 95 °C for 20 min. Endogenous peroxidase activity was quenched with 3% hydrogen peroxide for 10 min, and nonspecific binding was blocked with 5% normal goat serum for 1 h at room temperature. Sections were incubated with primary antibodies (anti-NatD or anti-FGFR4; see Supplementary Table 1) overnight at 4 °C, followed by incubation with HRP-conjugated secondary antibodies for 1 h. Immunoreactivity was

visualized using 3,3’-diaminobenzidine (DAB) and counterstained with hematoxylin. Slides were dehydrated, mounted, and imaged under a bright-field microscope (Olympus BX53). Immunoreactivity was scored using the H-score method: H-score = $\sum$ (Pi × i), where i is staining intensity (0–2) and Pi is the percentage of positive cells, categorized as 0 (0%), 1 (1–30%), 2 (31–60%), or 3 (61–100%). Immunoreactivity in mouse tumor tissues was quantified using ImageJ software by measuring staining intensity or the percentage of positive cells, as indicated. Positive and negative controls were included for antibody validation. The use of de-identified, commercially available human tissue arrays was exempt from IRB approval.

Cell culture

LN229 (CRL-2611) and U-87 MG (HTB-14) were obtained from the American Type Culture Collection (ATCC), and U-373 MG (KCLB 30017) was purchased from the Korean Cell Line Bank (Seoul, Korea). Cells were cultured in DMEM supplemented with 10% fetal bovine serum and 1% penicillin–streptomycin at 37 °C in a humidified 5% CO_2 incubator. Patient-derived GBM stem cells (PDC124 and PDC131) and stem-like derivatives of U-87 MG and U-373 MG cells were maintained in DMEM/F12 supplemented with epidermal growth factor (EGF, 10 ng/ml), basic fibroblast growth factor (bFGF, 5 ng/ml), B27 supplement, and 1% penicillin–streptomycin. All cell lines were routinely tested negative for mycoplasma contamination.

Lentiviral plasmid construction and stable cell line generation

NatD knockdown was achieved using short hairpin RNAs (shRNAs) [TRCN0000338552 (#2) and TRCN0000164279 (#4); Sigma-Aldrich] cloned into pLKO.1. NatD reconstitution was performed using pCDH-CMV-MCS-EF1-copGFP vectors encoding either wild-type NatD (NatD-WT) or a catalytically inactive mutant (E139Q, NatD-EQ). A FLAG peptide sequence was inserted at the C-terminus of NatD. For double knockdown experiments, shNatD and shCK2α (TRCN0000320926) were used. FGFR4 overexpression was achieved by lentiviral transduction of FGFR4 cDNA cloned

into pCDH-CMV-MCS-EF1-copGFP vector. Lentiviral particles were produced in HEK293T cells, concentrated, and used to infect GBM cells and GBM stem-like cells on fibronectin-coated wells. Stable cell lines were selected using puromycin (2 μg/ml) and successful transduction was verified by GFP fluorescence and immunoblot analysis.

Immunoblot details

Cells were lysed in RIPA buffer (50 mM Tris-HCl pH 8.0, 150 mM NaCl, 0.1% SDS, 0.5% sodium deoxycholate, 1% NP-40) supplemented with protease/phosphatase inhibitors. Lysates were clarified by centrifugation (13,000 rpm, 20 min). Protein concentrations were determined by Bradford assay. Equal amounts of protein were separated on 8–14% SDS-PAGE and transferred to PVDF membranes. Membranes were blocked with 5% (w/v) bovine serum albumin (BSA) for phospho-specific antibodies or 5% (w/v) non-fat dry milk in TBST, incubated overnight at 4 °C with primary antibodies (Supplementary Table 1), and then with HRP-conjugated secondary antibodies for 1 h. Protein bands were visualized using enhanced chemiluminescence (ECL).

Generation of the anti–Ac-N-H4 antibody

Polyclonal antibodies were generated in rabbits against an N-terminally acetylated histone H4 peptide (Ac-SGRGKGGKGLGK-Cys), in which an additional C-terminal cysteine residue was introduced for conjugation. The peptide was conjugated to maleimide-activated keyhole limpet hemocyanin (mcKLH; Thermo Fisher Scientific, #77605) via thiol–maleimide coupling. Rabbits were immunized with the antigen at 10–14 day intervals for a total of 10 immunizations, and whole blood was collected after the final boost. Antibodies were affinity-purified using peptide-conjugated SulfoLink resin (Thermo Fisher Scientific, #20401) according to the manufacturer’s instructions.

Validation of the anti-Ac-N-H4 antibody

The specificity of the custom anti–Ac-N-H4 antibody was validated using dot blot, peptide immunoblot, and peptide competition assays, as described below.

Dot blot and peptide immunoblot analyses

Histone H4 peptides (H4: SGRGKGGKGLGKGGAKRHRK-biotin; Ac-H4: Ac-SGRGKGGKGLGKGGAKRHRK-biotin) were spotted onto nitrocellulose membranes at the indicated amounts and air-dried. Membranes were blocked with 5% (w/v) non-fat dry milk in TBST for 1 h at room temperature and incubated with primary antibodies overnight at 4 °C. The following primary antibodies were used: anti-H4 and a custom-made anti–Ac-N-H4 antibody. After washing with TBST, membranes were incubated with HRP-conjugated secondary antibodies for 1 h at room temperature. Signals were detected using enhanced chemiluminescence (ECL) according to the manufacturer's instructions.

For peptide immunoblot analysis, histone H4 peptides were resolved by SDS–PAGE under denaturing conditions and transferred to nitrocellulose membranes, followed by immunodetection with the anti–Ac-N-H4 antibody. Peptide loading was verified by Coomassie staining.

Peptide competition assay

For peptide competition assays, the anti–Ac-N-H4 antibody was pre-incubated with N-terminally acetylated H4 peptide (Ac-H4; 100 μg/ml) or unmodified H4 peptide (H4; 100 μg/ml) for 1 h at room temperature prior to immunoblotting. The antibody–peptide mixtures were then applied to membranes, and immunoblotting was performed as described above.

Functional assays

For proliferation assays, cells were seeded at the appropriate density and counted at 24 h intervals (GBM cell lines) or 48 h intervals (GSCs) using trypan blue exclusion. Colony formation assays were performed by seeding 500 cells per well in 6-well plates, followed by fixation and crystal violet

staining after 14 days. Cell migration was assessed by wound-healing assay, and invasion was evaluated using Matrigel-coated transwell chambers (8 μm-pores). For wound-healing assays, cells were seeded in 6-well plates and cultured to confluence. A linear wound was created using a sterile pipette tip, followed by washing with PBS to remove detached cells. Subsequently, fresh medium was added, and images were captured at 0 h and 24 h to minimize the contribution of cell proliferation. Wound closure was quantified using ImageJ software by measuring the wound area, and migration was expressed as the percentage of wound width relative to the initial wound area.

Extreme limiting dilution assay (ELDA)

GBM stem-like cells (GSCs) of U-87 MG and U-373 MG cells, and patient-derived GBM stem cells were seeded at 200, 100, 50, and 25 cells per well in 96-well ultra-low attachment plates in serum-free DMEM/F12 medium containing B27, EGF (10 ng/ml), and bFGF (5 ng/ml). Sphere formation was monitored for 14 days, and wells containing single tumorspheres were scored as positive. Stem cell frequency was calculated using the ELDA software (http://bioinf.wehi.edu.au/software/elda/).

RNA sequencing and bioinformatics

Total RNA from LN229 cells stably expressing control shRNA (shCon), shRNA targeting NatD (shNatD) with lentiviral empty vector, and shNatD with lentiviral wild-type NatD construct (shNatD/NatD-WT) was extracted and quality-checked using Qubit and TapeStation. Libraries were prepared using TruSeq RNA Library Prep Kit v2 and sequenced on Illumina NovaSeq 6000 (101-bp paired-end). Reads were trimmed (Trimmomatic), aligned (STAR v2.7.8a), and quantified (HTSeq, GENCODE v41). DEGs were identified using edgeR and SARTools (FDR < 0.05, fold change ≥ 1.5). Gene Ontology (GO) and pathway enrichment were analyzed with Metascape. Details of the transcriptome data are presented in Supplementary Table 4–6, and data are available in GEO (accession: GSE312750).

Quantitative real-time RT-PCR (qRT-PCR)

qRT-PCR was performed with SensiMix SYBR Hi-ROX (Meridian Bioscience) on a StepOnePlus system under standard conditions (95 °C 30 sec; 40 cycles of 95 °C 5 sec and 60 °C 30 sec). Relative expressions were calculated by the $2^{-\Delta\Delta Ct}$ method using β-actin or GAPDH as normalizers[1]. Primer sequences are listed in Supplementary Table 2.

Chromatin immunoprecipitation (ChIP) assay

Cells were crosslinked with 1% formaldehyde for 10 min and quenched with 125 mM glycine for 10 min. Chromatin was sonicated to 200–500 bp fragments and immunoprecipitated overnight at 4 °C with Protein G Sepharose beads (Cytiva) using primary antibodies listed in Supplementary Table 1. Normalization used pan-H3 and pan-H4, and IgG served as a negative control. After sequential washes with IP washing buffer and TE buffer, chromatin was eluted using elution buffer containing SDS and $NaHCO_3$ and then reverse-crosslinked by overnight incubation with NaCl and proteinase K at 65 °C. DNA was purified using a PCR purification kit (Qiagen) and analyzed by quantitative real-time PCR (qPCR) with primers targeting the FGFR4 promoter regions (P1 and P2, Fig. 6a) or a negative control region located ~20 kb downstream of the FGFR4 gene, predicted as a gene-independent site by UniBind (https://unibind.uio.no/).

Measurement of NatD enzymatic activity (*in vitro* acetylation assay)

The N-terminal acetyltransferase activity of NatD was measured by *in vitro* acetylation assay based on previous reports[2,3]. Recombinant His-tagged NatD-WT and NatD-EQ proteins were expressed in *E. coli* BL21 (DE3, Real Biotech, Cat. No. RH217) and purified using Ni-NTA affinity chromatography (Ni-NTA Agarose, Qiagen, Cat. No. 30210). Synthetic histone H4 N-terminal peptides (NonAc-H4: SGRGKGGKGLGKGGAK; Ac-H4: Ac-SGRGKGGKGLGKGGAK; GL Biochem, Shanghai, China; purity >90%) were used as substrates. Reactions containing 300 nM enzymes, 300 μM peptides, and 300 μM acetyl-CoA (MP Biomedicals, Cat. No. 02100490-CF) in acetylation buffer (100 mM

HEPES-HCl pH 7.5, 200 mM NaCl, 2 mM EDTA) were incubated at 37 °C for 30 min. Reactions were quenched with quenching buffer (3.2 M guanidinium-HCl, 100 mM sodium phosphate dibasic pH 6.8), mixed with DTNB buffer (100 mM sodium phosphate dibasic pH 6.8, 10 mM EDTA, 10 mg/ml DTNB; Thermo Fisher Scientific, Cat. No. 22582), and absorbance was immediately measured at 412 nm using a 96-well plate reader. TNB formation was quantified according to Beer–Lambert's law as previously described[2,3].

Orthotopic xenograft models

Six-week-old male BALB/c nude mice were used for all experiments. LN229 cells (2×10^5 cells) were stereotactically implanted into the striatum using a Hamilton syringe under anesthesia. Mice were monitored daily and euthanized at the experimental endpoint or when humane endpoint criteria were met.

Magnetic resonance imaging (MRI) analysis

Magnetic resonance imaging (MRI) was performed at 5 weeks post-implantation using a 9.4 T MRI scanner (Bruker BioSpin GmbH). Mice were anesthetized with isoflurane during imaging. Tumor volumes were quantified using 3D Slicer software (http://www.slicer.org).

Survival analysis

For survival analysis, mice (n = 5 per group) were monitored until death or until humane endpoint criteria were reached. Survival curves were generated using the Kaplan–Meier method and statistically analyzed using the log-rank (Mantel–Cox) test.

References

1. Schmittgen, T.D. & Livak, K.J. Analyzing real-time PCR data by the comparative C(T) method. *Nat Protoc*. **3**, 1101–1108 (2008).
2. Foyn, H., Thompson, P.R. & Arnesen, T. DTNB-based quantification of in vitro enzymatic n-terminal acetyltransferase activity. *Methods Mol Biol*. **1574**, 9–15 (2017).
3. Jung, T.Y. et al. Naa20, the catalytic subunit of NatB complex, contributes to hepatocellular carcinoma by regulating the LKB1–AMPK–mTOR axis. *Exp Mol Med*. **52**, 1831–1844 (2020).

Supplementary Figures

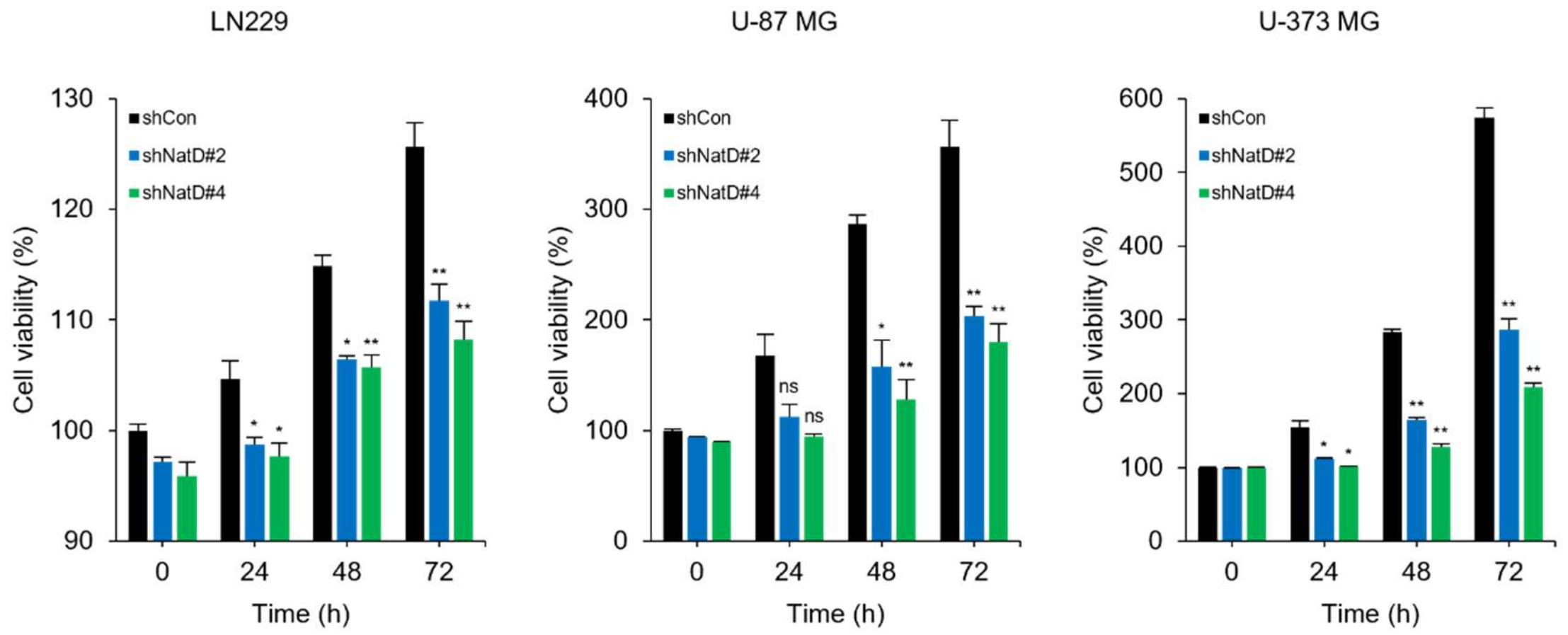

Supplementary Figure 1. NatD knockdown reduces cell viability in GBM cell lines.

Cell viability was assessed over 72 h following NatD knockdown in three GBM cell lines: LN229, U-87 MG, and U-373 MG. Cells were transfected with control shRNA (shCon) or two independent shRNAs targeting NatD (shNatD#2 and shNatD#4), and viability was measured using an MTS assay (CellTiter 96 Aqueous One Solution, Cat. No. G3580; Promega). NatD knockdown significantly reduced cell viability compared to control, with the most pronounced effects observed at 72 h.

Data represent mean ± SEM from three independent experiments. Statistical significance was determined using one-way ANOVA. *$P < 0.05$, **$P < 0.01$; ns, not significant.

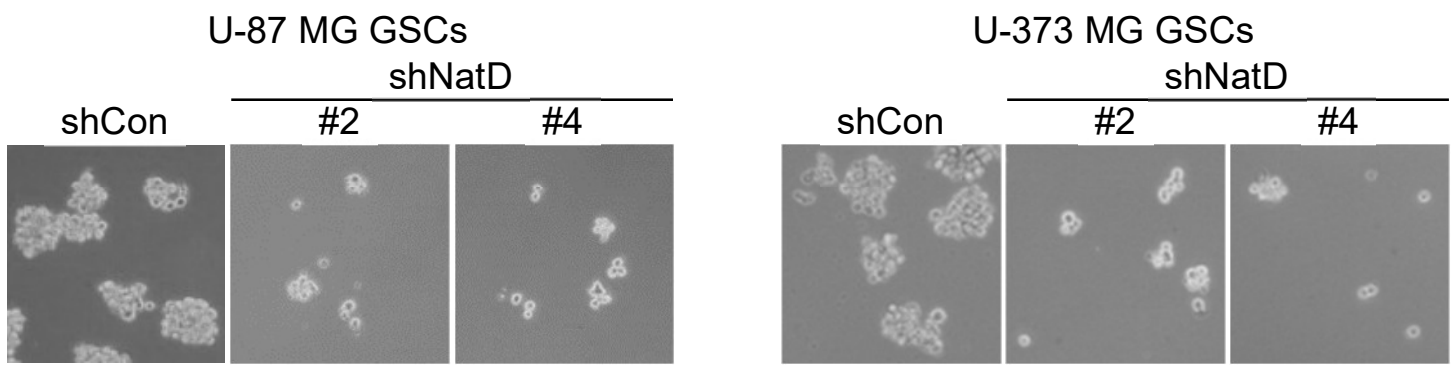

Supplementary Figure 2. NatD knockdown suppresses tumorsphere formation in GBM stem-like cells (GSCs).

Tumorsphere formation assays were performed using GBM stem-like cells (GSCs) derived from U-87 MG and U-373 MG cells. Cells were stably expressing control shRNA (shCon) or NatD-targeting shRNAs (shNatD#2 and shNatD#4). Representative images show that NatD knockdown markedly reduced both the number and size of tumorspheres compared to control. Images are representative of three independent experiments.

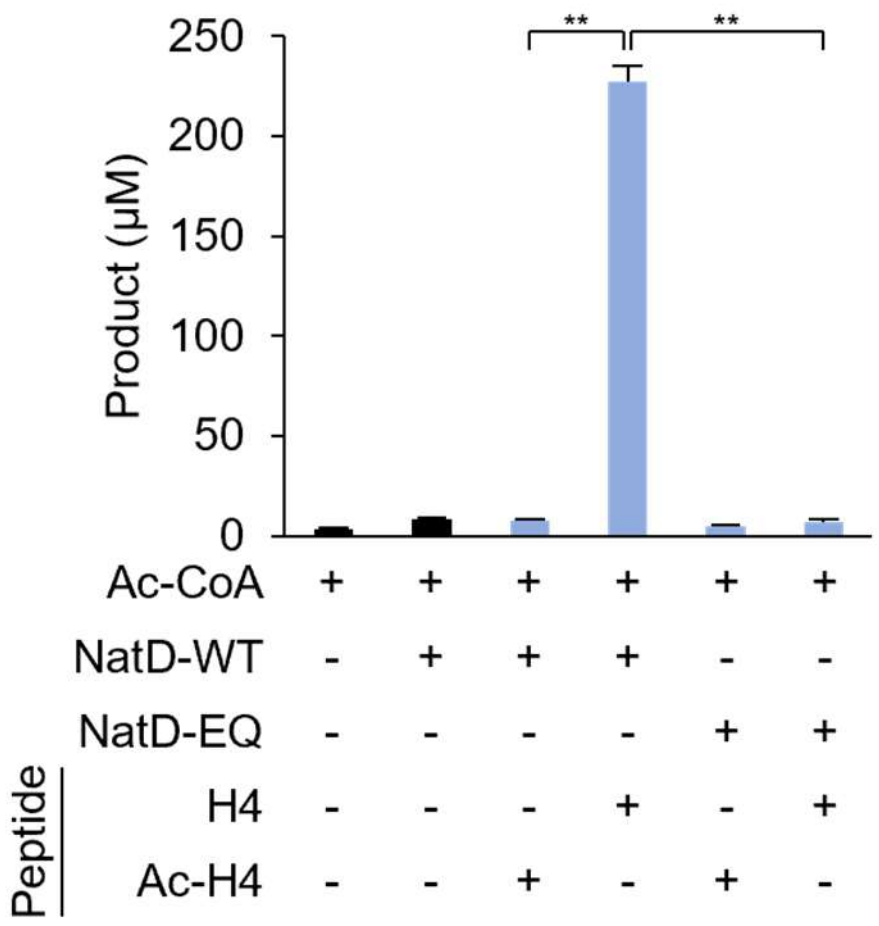

Supplementary Figure 3. Measurement of NatD enzymatic activity.

N-terminal acetyltransferase activity of wild-type NatD (NatD-WT) and an enzymatically inactive mutant (NatD-EQ) was measured using DTNB-based *in vitro* acetylation assay. The assay was performed with NatD-WT or NatD-EQ in the presence of NonAc-H4 or Ac-H4 peptides. Enzymatic activity was significantly increased only in NatD-WT with NonAc-H4, whereas no increase was observed in NatD-EQ with NonAc-H4. Data are presented as mean ± SEM (n = 3). Statistical significance was determined using one-way ANOVA followed by Tukey's multiple comparison test. $^{**}P < 0.01$.

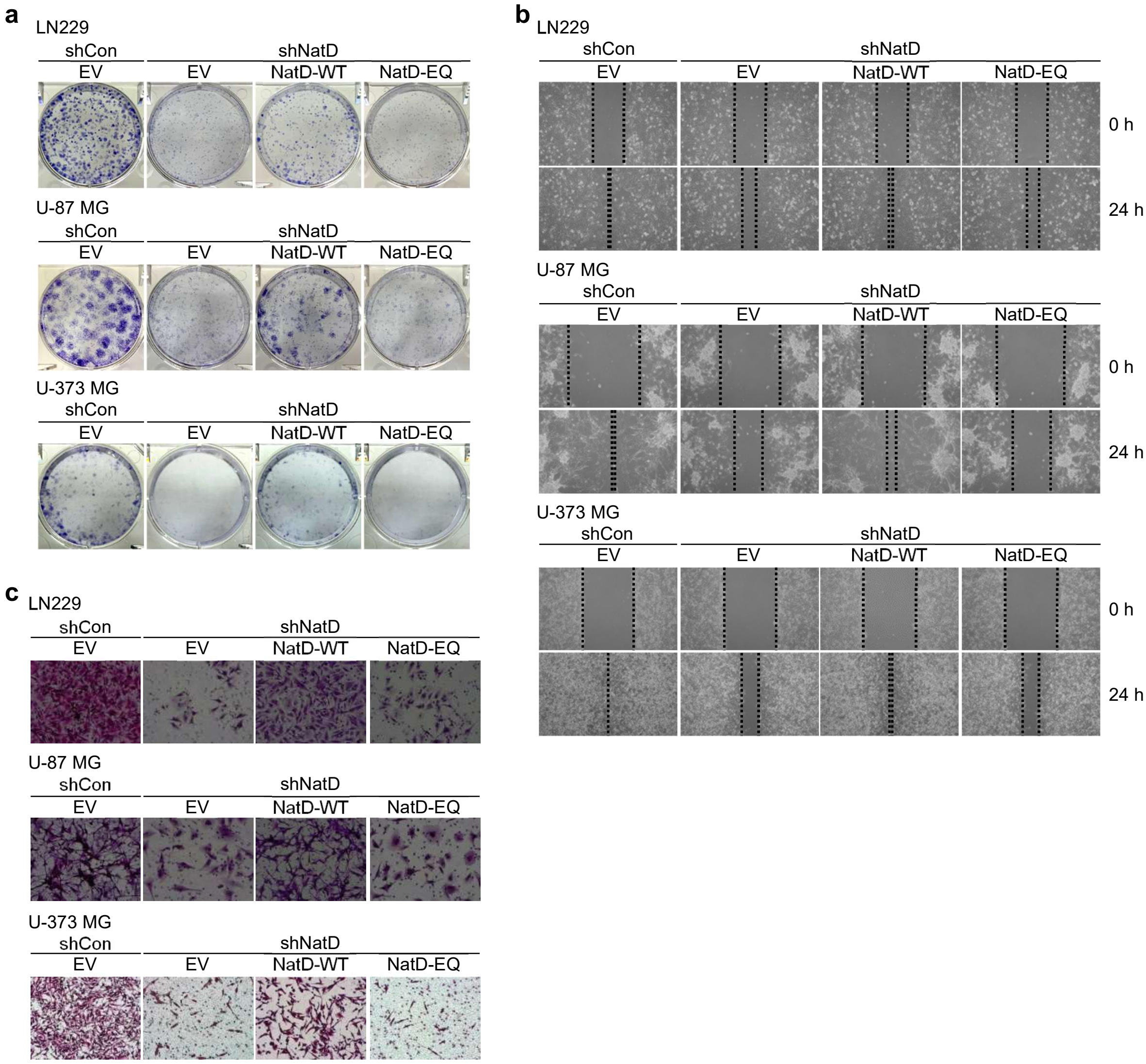

Supplementary Figure 4. Enzymatic activity of NatD is required for its oncogenic functions in GBM cells.

a Colony formation assays in LN229, U-87 MG, and U-373 MG cells following NatD knockdown (shNatD) and rescue with either wild-type NatD (NatD-WT) or an enzymatically inactive mutant (NatD-EQ). shCon- and shNatD-expressing cells were transduced with the corresponding empty lentiviral vector to ensure equivalent viral exposure and selection conditions across all groups. NatD-WT restored colony formation, whereas NatD-EQ failed to do. **b** Wound healing assays performed at 24 h under the same conditions. NatD-WT rescued migratory capacity, while NatD-EQ did not. **c** Transwell invasion assays showing that NatD-WT, but not NatD-EQ, restored invasive ability in NatD-depleted cells. Representative images from three independent experiments are shown.

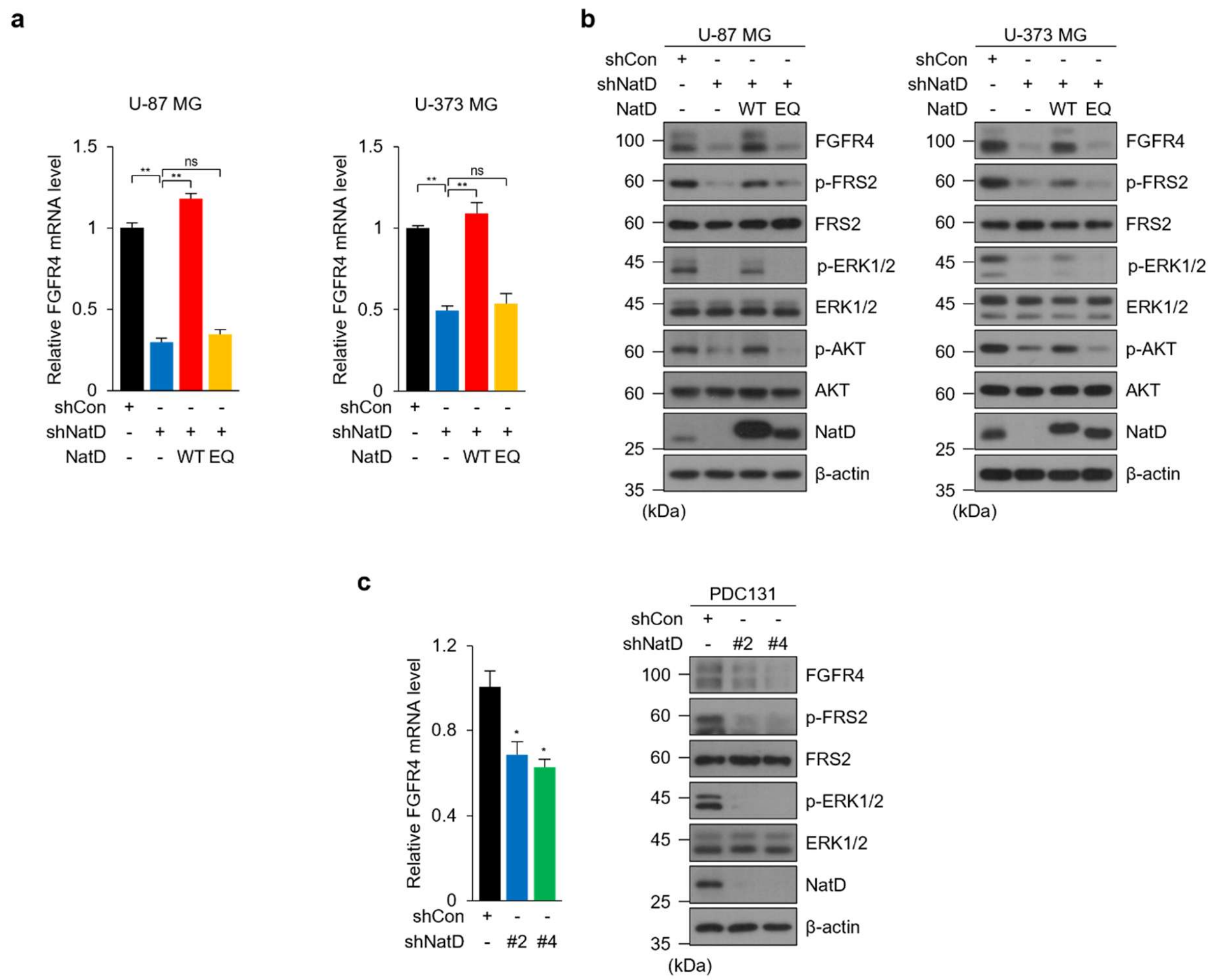

Supplementary Figure 5. FGFR4 is a transcriptional target of NatD.

a–**b** U-87 MG and U-373 MG cells stably expressing control shRNA (shCon) with empty vector, shRNA targeting NatD (shNatD) with empty vector, shNatD with NatD-WT (wild-type) or shNatD with NatD-EQ (a catalytically inactive mutant) were subjected to qRT-PCR analysis of FGFR4 expression (**a**) and immunoblot analysis of the indicated proteins (**b**). **c** PDC131 stably expressing control shRNA (shCon) or shRNAs targeting NatD (shNatD#2, shNatD#4) were subjected to qRT-PCR analysis (left) and immunoblot analysis (right). β-actin mRNA was used as an internal control for qRT-PCR, and β-actin protein served as a loading control for immunoblot analysis. Representative images and quantification of three independent experiments are shown. Data are presented as mean ± SEM. Statistical significance was determined using one-way ANOVA followed by Tukey's multiple comparison test. **P* < 0.05, ***P* < 0.01; ns, not significant.

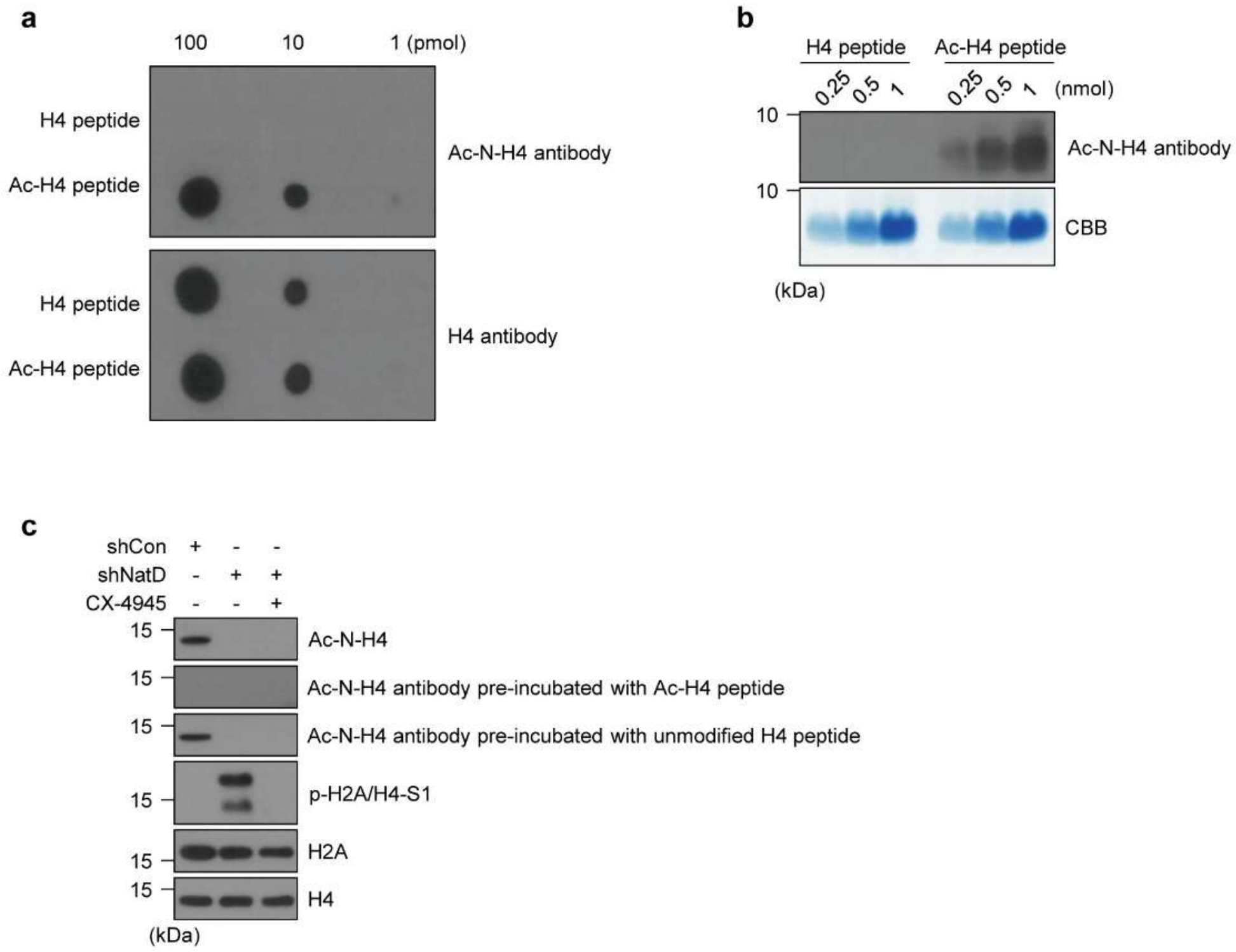

Supplementary Figure 6. Specificity of the anti–Ac-N-H4 antibody.

a Dot blot analysis of antibody specificity using histone H4 peptides. Indicated amounts of unmodified H4 and N-terminally acetylated H4 (Ac-H4) peptides were spotted onto a nitrocellulose membrane and probed with pan-H4 or Ac-N-H4 antibodies. The pan-H4 antibody detected both unmodified H4 and Ac-H4 peptides, whereas the Ac-N-H4 antibody selectively recognized only the Ac-H4 peptide, with no detectable cross-reactivity toward the unmodified H4 peptide. **b** Peptide immunoblot analysis of histone H4 peptides. Indicated amounts of unmodified H4 and Ac-H4 peptides were separated by electrophoresis, transferred onto a nitrocellulose membrane, and probed with the Ac-N-H4 antibody. Peptide loading was confirmed by Coomassie staining. **c** LN229 cells stably expressing control shRNA (shCon) or shNatD#4 (shNatD) were treated with vehicle or CX-4945 (20 μM) for 24 h and subjected to immunoblot analysis of the indicated proteins. For peptide competition assays, the Ac-N-H4 antibody was pre-incubated with Ac-H4 peptide or unmodified H4 peptide prior to immunoblotting. Pre-incubation with Ac-H4 peptide abolished the Ac-H4 signal, whereas pre-incubation with unmodified H4 peptide had no effect. H2A and H4 served as loading controls.

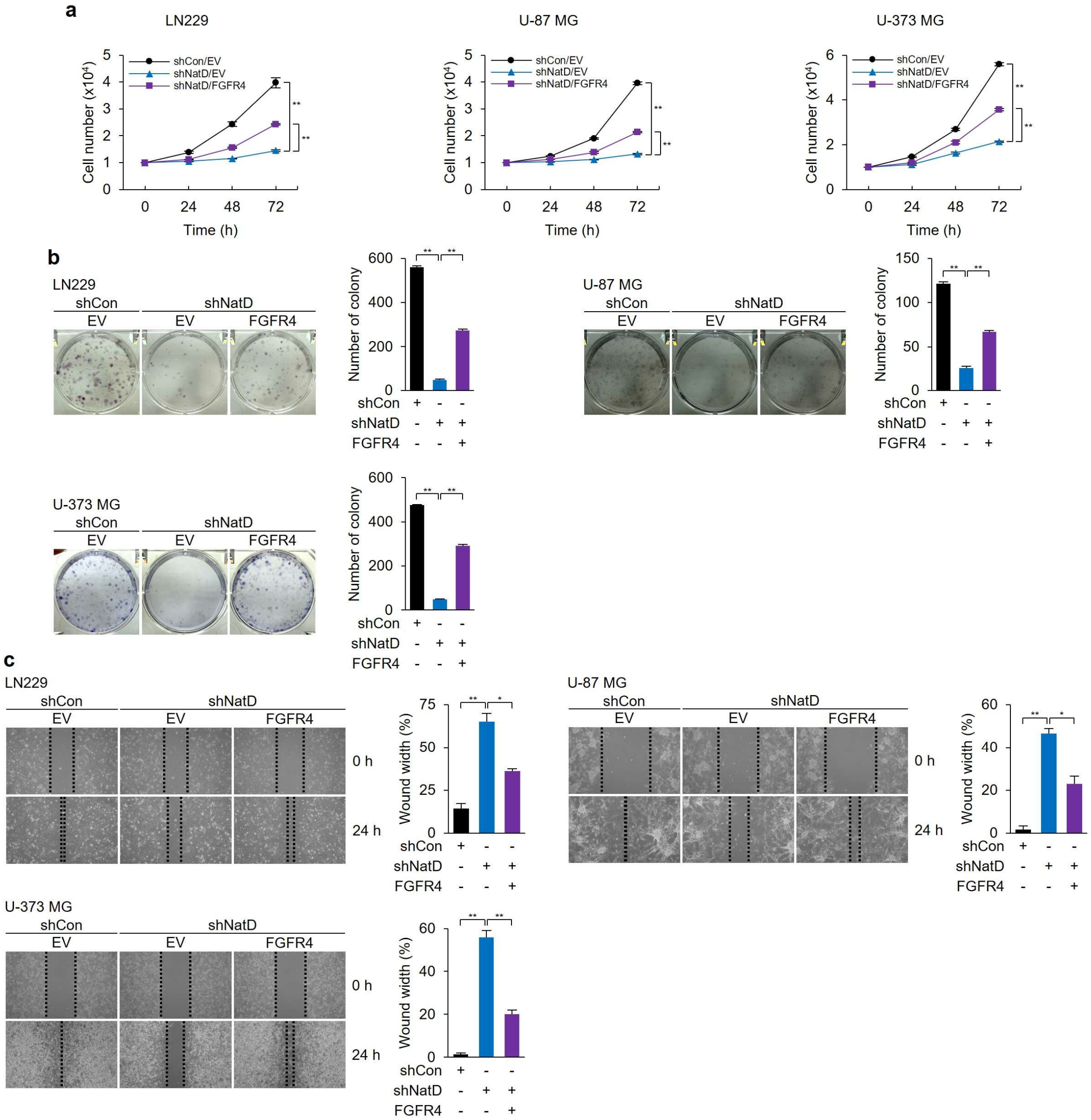

a
LN229
U-87 MG
U-373 MG
Cell number (x10⁴)
shCon/EV
shNatD/EV
shNatD/FGFR4
Time (h)
0
24
48
72
**
b
LN229
shCon
shNatD
EV
EV
FGFR4
Number of colony
shCon + - -
shNatD - + +
FGFR4 - - +
U-87 MG
U-373 MG
c
LN229
shCon
shNatD
EV
EV
FGFR4
0 h
24 h
Wound width (%)
shCon + - -
shNatD - + +
FGFR4 - - +
U-87 MG
U-373 MG

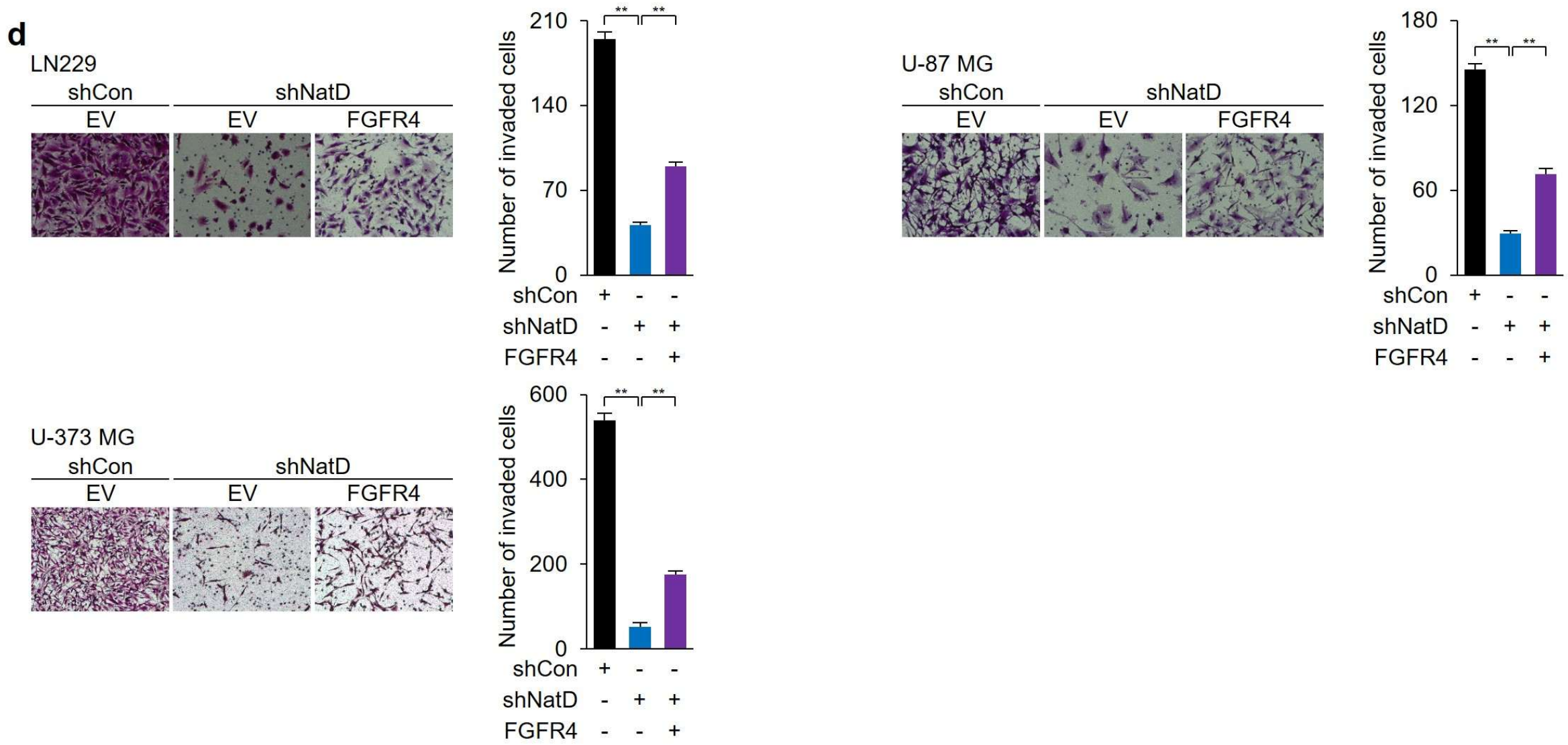

Supplementary Figure 7. FGFR4 overexpression rescues the impaired growth, migration, and invasion caused by NatD knockdown in GBM cells.

GBM cell lines (LN229, U-87 MG, and U-373 MG) stably expressing control shRNA with empty vector (shCon/EV), NatD-targeting shRNA with empty vector (shNatD/EV), or shNatD with lentivirus overexpressing FLAG-FGFR4 (shNatD/FGFR4) were used.

a Cell counting assays performed every 24 h for up to 72 h. FGFR4 overexpression restored cell proliferation suppressed by NatD knockdown. **b** Colony formation assays showing that FGFR4 overexpression rescued clonogenic growth in NatD-depleted cells. **c** Wound healing assays at 0 and 24 h demonstrating that FGFR4 overexpression recovered migratory capacity impaired by NatD knockdown. **d** Transwell invasion assays showing that FGFR4 overexpression restored invasive ability in NatD-depleted cells. Representative images and quantification of three independent experiments are shown. Data are presented as mean ± SEM. Statistical significance was determined using one-way ANOVA followed by Tukey's multiple comparison test. $^{**}P < 0.01$.

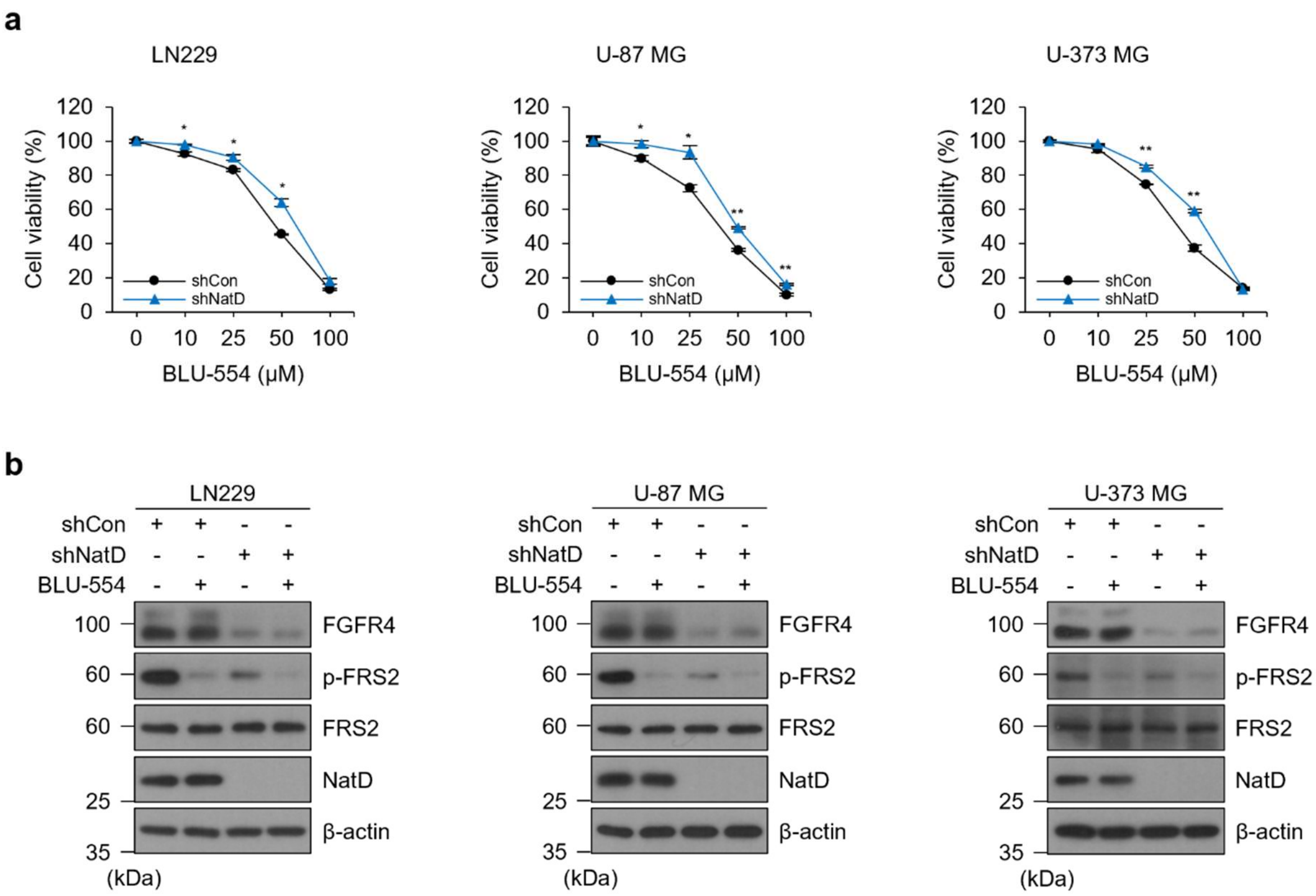

Supplementary Figure 8. NatD knockdown attenuates the anti-proliferative effect of the FGFR4 inhibitor, BLU-554.

LN229, U-87 MG, and U-373 MG cells stably expressing control shRNA (shCon) or shRNA targeting NatD (shNatD) were treated with indicated concentrations of BLU-554 for 48 h. Cell viability was measured using an MTS assay (**a**) and immunoblot analysis of the indicated proteins was performed (**b**). β-actin was used as a loading control. Data are presented as mean ± SEM of three independent experiments. Statistical significance was determined using one-way ANOVA. **P* < 0.05, ***P* < 0.01.

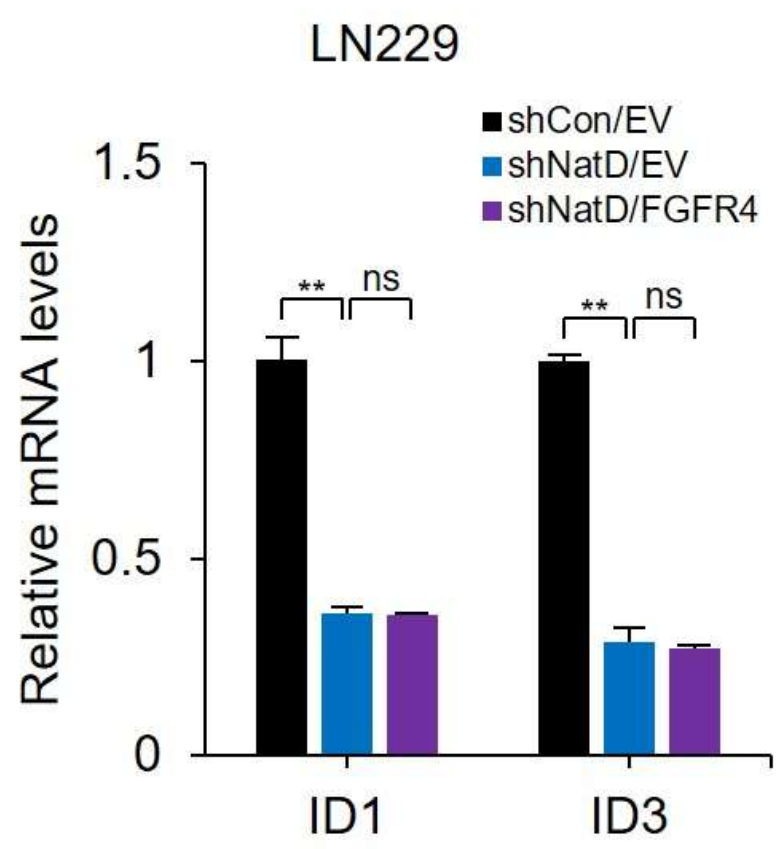

Supplementary Figure 9. FGFR4 overexpression does not restore TGF-β target gene expression following NatD depletion.

LN229 cells stably expressing control shRNA with empty vector (shCon/EV), NatD-targeting shRNA with empty vector (shNatD/EV) or shNatD with FLAG-FGFR4 (shNatD/FGFR4) were subjected to qRT-PCR analysis of ID1 and ID3 using β-actin as an internal control. Data are presented as mean ± SEM of three independent experiments. Statistical significance was determined using one-way ANOVA followed by Tukey's multiple comparison test. $^{**}P < 0.01$; ns, not significant.

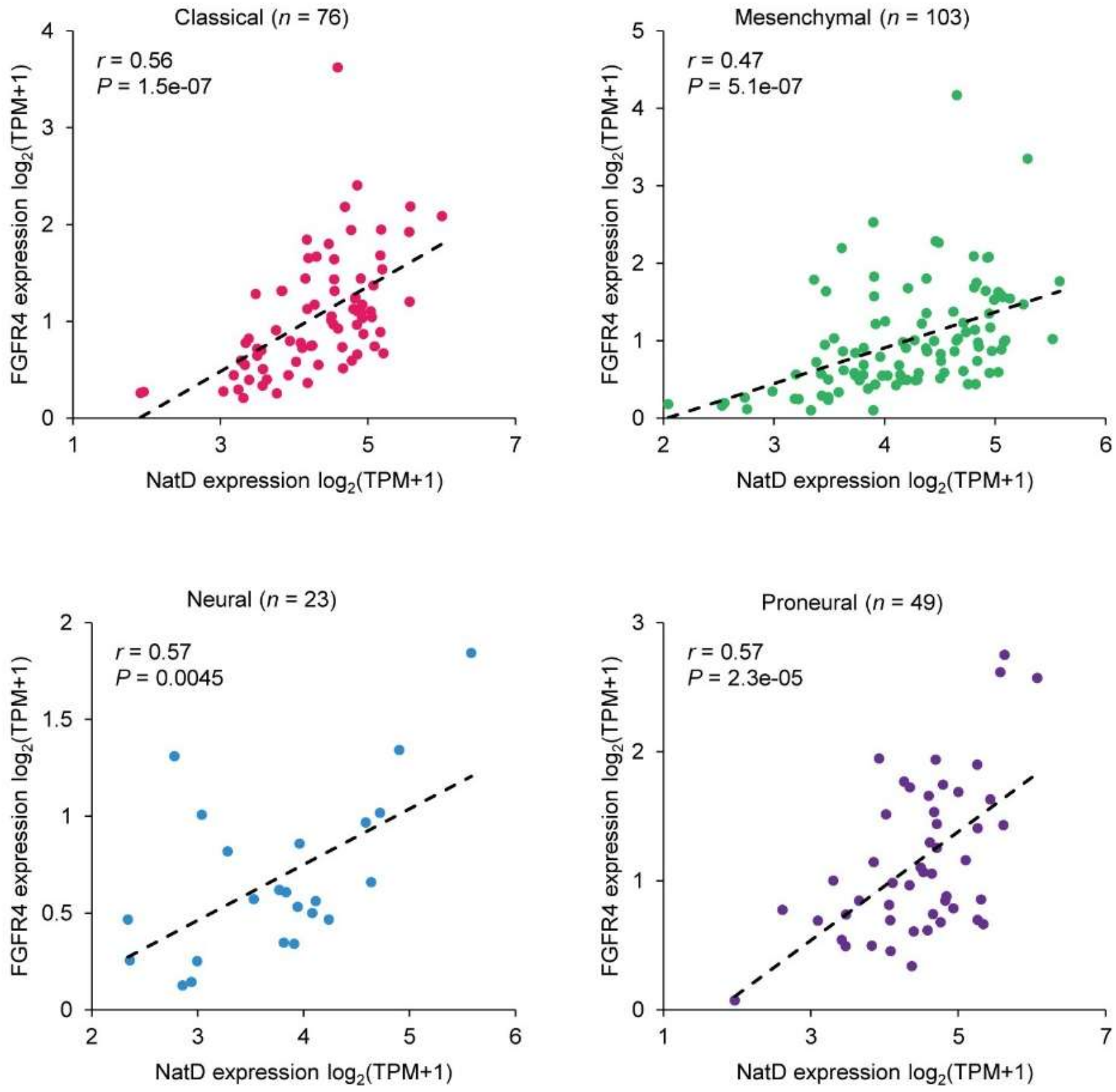

Supplementary Figure 10. Subtype-stratified correlation of the NatD–FGFR4 axis in TCGA GBM.

Correlation analyses between NatD and FGFR4 mRNA expression were performed using TCGA–GBM RNA sequencing data ($n = 251$). Samples were stratified into molecular subtypes (Classical, Mesenchymal, Neural, and Proneural), and scatter plots show the relationship between NatD and FGFR4 expression in each subtype. Expression values are presented as $\log_2(\mathrm{TPM} + 1)$. Correlation coefficients (r) and statistical significance (P values) were determined using Spearman correlation analysis. A positive correlation between NatD and FGFR4 expression was observed across all molecular subtypes, indicating that the NatD–FGFR4 axis is broadly conserved in GBM. The corresponding Spearman correlation coefficients (r) and P values are indicated in each panel.

Supplementary Tables

List

Supplementary Table 1. Antibodies used in this study

Supplementary Table 2. Primers used in this study

Supplementary Table 3. Stem cell frequencies

Supplementary Table 4. Description of whole-transcriptome sequencing data

Supplementary Table 5. Differentially expressed genes (DEGs) among shControl, shNatD, and shNatD/NatD-WT groups

Supplementary Table 6. Enriched Gene Ontology terms and pathways for 391 NatD-dependent DEGs

Supplementary Tables 1, 2, and 3 are included in this Supplementary Information file and Supplementary Tables 4, 5, and 6 are provided as separate Excel files.

Supplementary Table 1. Antibodies used in this study

Target protein	Host species	Clone / Type	Vendor (Company)	Catalog No.	Application (WB/IHC/ChIP)	Dilution
NatD (NAA40)	Rabbit	Polyclonal	Abcam	ab106408	IHC	1:200
NatD (NAA40)	Rabbit	Polyclonal	Abclon	Custom-made	WB	1:1000
FGFR4	Mouse	Monoclonal	Biorbyt	orb69074	IHC	1:1600
FGFR4	Rabbit	Monoclonal	Cell Signaling Technology	8562	WB	1:1000
FRS2	Rabbit	Polyclonal	Invitrogen	PA5-27875	WB	1:1000
p-FRS2 (Tyr436)	Rabbit	Polyclonal	Invitrogen	PA5-37785	WB, IHC	1:1000 (WB) 1:200 (IHC)
ERK1/2	Rabbit	Polyclonal	CST	9102	WB	1:1000
p-ERK1/2 (Thr202/Tyr204)	Rabbit	Polyclonal	CST	9101	WB	1:1000
	Mouse	Monoclonal	Invitrogen	14-9109-82	IHC	1:200
AKT	Rabbit	Polyclonal	CST	9272	WB	1:1000
p-AKT (Ser473)	Rabbit	Polyclonal	CST	9271	WB	1:1000
Ki-67	Rabbit	Monoclonal	Invitrogen	MA5-14520	IHC	1:200
Nestin	Mouse	Monoclonal	Abcam	ab6320	WB	1:1000
OCT4	Rabbit	Monoclonal	CST	2840	WB	1:1000
SOX2	Goat	Polyclonal	Abcam	ab239218	WB	1:1000
H2A	Rabbit	Polyclonal	Abcam	ab18255	WB	1:1000
H3	Rabbit	Polyclonal	Abcam	ab1791	ChIP	2 μg
H4	Rabbit	Polyclonal	Abcam	ab10158	WB, ChIP	1:1000 (WB), 2 μg (ChIP)
Ac-N-H4	Rabbit	Polyclonal		Custom-made	WB, ChIP	1:1000 (WB), 2 μg (ChIP)
p-H2A/H4-S1	Rabbit	Monoclonal	Abcam	ab177309	WB, ChIP	1:1000 (WB), 2 μg (ChIP)
H3K4me3	Rabbit	Polyclonal	Merck Millipore	07-473	ChIP	2 μg
H3K27me3	Rabbit	Polyclonal	Active Motif	39155	ChIP	2 μg
CK2α	Rabbit	Polyclonal	Abclonal	A1014	WB	1:1000
β-actin	Mouse	Monoclonal	Santa Cruz	sc-47778	WB	1:1000
Secondary (anti-Rabbit HRP)	Goat	Polyclonal	Invitrogen	A16104	WB, IHC	1:5000 (WB), 1:500 (IHC)

Secondary (anti-Mouse HRP)	Goat	Polyclonal	Invitrogen	A16072	WB, IHC	1:5000 (WB), 1:500 (IHC)

Supplementary Table 2. Primers used in this study

Gene	Forward primer (5'→3')	Reverse primer (5'→3')	Application
FGFR4	GAGGGGCCGCCTAGAGATT	CAGGACGATCATGGAGCCT	qPCR
β-actin	AGAGCTACGAGCTGCCTGAC	AGCACTGTGTTGGCGTACAG	qPCR (housekeeping)
GAPDH	AGCCACATCGCTCAGACAC	GCCCAATACGACCAAATCC	qPCR (housekeeping)
FGFR4 promoter (P2)	TGGGTGGGGCGAAAGAGGCG	GGGGTGGCCAAGATCCCCGC	ChIP-qPCR
FGFR4 promoter (P1)	TGGCCACACCTCTCTCCGGC	TGCACGCGGCAGGGTCCGCA	ChIP-qPCR
Negative control (non-binding)	ATGGCTAAACGCCGTCTCTA	GTGGCACAATCTCACTGCAA	ChIP-qPCR

Supplementary Table 3. Stem cell frequencies

	Cells	Group	Estimate (Range)
Figure 3b	U-87 MG GSCs	shCon	1 in 13 (8-20)
		shNatD#2	1 in 194 (133-284)
		shNatD#4	1 in 278 (181-428)
	U-373 MG GSCs	shCon	1 in 51 (37-71)
		shNatD#2	1 in 236 (157-354)
		shNatD#4	1 in 297 (191-461)
Figure 3d	PDC124	shCon	1 in 13 (8-20)
		shNatD#2	1 in 29 (21-41)
		shNatD#4	1 in 29 (21-41)
	PDC131	shCon	1 in 30 (21-42)
		shNatD#2	1 in 398 (243-652)
		shNatD#4	1 in 431 (259-718)
Figure 4e	U-87 MG GSCs	shCon	1 in 22 (15-31)
		shNatD/Vector	1 in 263 (173-402)
		shNatD/NatD-WT	1 in 77 (56-105)
		shNatD/NatD-EQ	1 in 260 (171-396)
	U-373 MG GSCs	shCon	1 in 28 (20-40)
		shNatD/Vector	1 in 247 (164-374)
		shNatD/NatD-WT	1 in 89 (64-122)
		shNatD/NatD-EQ	1 in 293 (189-454)
Figure 7b	U-87 MG GSCs	shCon	1 in 23 (16-33)
		shNatD/Vector	1 in 326 (207-515)
		shNatD/FGFR4	1 in 86 (63-119)

Stem cell frequencies were calculated using ELDA software based on sphere formation assays shown in Figures 3b, 3d, 4e, and 7b. Values are presented as estimated stem cell frequency (1 in *n*) with 95% confidence intervals in parentheses. Analyses were performed using two independent shRNAs targeting NatD where indicated or shNatD#4 where not specified, as described in the corresponding figure legends. Values are calculated from at least three independent experiments.
